# Using Ongoing Wildlife Surveillance Programs to Monitor Antibiotic Resistant Bacteria in the Environment

**DOI:** 10.1111/1758-2229.70404

**Published:** 2026-08-16

**Authors:** María Rincón‐Gracia, Stefan Börjesson, Robert Söderlund, Hanna Woksepp, Aleksija Neimanis, Henrik Uhlhorn, Thomas Rosendal, David Winlund, Hugo Uddströmer, Josef D. Järhult, Jonas Bonnedahl

**Affiliations:** ^1^ Department of Animal Health and Antimicrobial Strategies Swedish Veterinary Agency Uppsala Sweden; ^2^ Department of Biomedical and Clinical Sciences Linköping University Linköping Sweden; ^3^ Department of Animal Health Norwegian Veterinary Institute Ås Norway; ^4^ Department of Microbiology Swedish Veterinary Agency Uppsala Sweden; ^5^ Department of Clinical Sciences Swedish University of Agricultural Sciences Uppsala Sweden; ^6^ Department of Clinical Microbiology Region Kalmar County Kalmar Sweden; ^7^ Department of Pathology and Wildlife Diseases Swedish Veterinary Agency Uppsala Sweden; ^8^ Department of Animal Biosciences Swedish University of Agricultural Sciences Uppsala Sweden; ^9^ Department of Epidemiology Swedish Veterinary Agency Uppsala Sweden; ^10^ Clinical Department of Surgery and Gastroenterology Malmö Sweden; ^11^ Region Dalarna, Mora lasarett Mora Sweden; ^12^ Department of Medical Sciences Uppsala University Uppsala Sweden; ^13^ Department of Infectious Diseases Region Kalmar County Kalmar Sweden

**Keywords:** antibiotic resistance, molecular epidemiology, surveillance, wildlife

## Abstract

The need to include wildlife in surveillance programs for antibiotic resistance has been previously highlighted. In the present study, we explored the possibility of sampling Swedish wildlife in conjunction with ongoing monitoring programs for wildlife health and disease to evaluate the occurrence of antibiotic resistance. The animals included in the study represented different trophic levels and degrees of interaction with humans, namely bears, eagles, wolves, foxes, otters, and hares. Samples were screened for ESBL‐, pAmpC‐, and carbapenemase‐producing *Escherichia coli* using selective media, and indicator *E. coli* was also isolated from each sample. All isolates were tested for antimicrobial susceptibility, and a subset of isolates was genome sequenced. Two samples from foxes were found to carry ESBL‐ or pAmpC‐producing isolates, while no carbapenemase‐producing *E. coli* could be detected. Geographical information on the samples and genetic characterization of the ESBL‐ and pAmpC‐producing isolates suggest that proximity to anthropogenic settings could play a role in the occurrence of antibiotic resistance in wildlife. In addition, the study demonstrated that the use of ongoing wildlife surveillance programs presents a cost‐efficient possibility to monitor antibiotic resistance in wild animals. However, future studies and monitoring programs should take careful consideration regarding selection of animal species and distribution.

## Graphical abstract

Screening of ESBL‐, pAmpC‐ and carbapenemase‐producing *Escherichia coli* among Swedish wildlife demonstrated a low occurrence of antibiotic resistance in wild animals. Only two ESBL/pAmpC‐producers were detected in red foxes originating from highly populated areas. Using ongoing wildlife surveillance programs presents a cost‐efficient possibility to monitor antibiotic resistance in the environment.

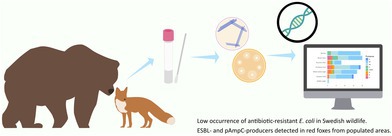

## 1 Introduction

Even though free‐ranging wildlife are not treated with antibiotics, it has been demonstrated that diverse wildlife species are carriers of antibiotic‐resistant bacteria (ARB) (Allen et al. 2010; Laborda et al. 2022). Proximity to anthropogenic settings and foraging habits seem to influence this occurrence, with wildlife living in proximity to livestock, feeding on carcasses, or visiting landfills and wastewater treatment plants more commonly carrying ARB (Sharma et al. 2018; Ahlstrom et al. 2021; Woksepp et al. 2023; Höcketstaller et al. 2025). While this indicates a form of anthropogenic pollution, it also raises concern, as wild animals might accumulate and disseminate ARB (Atterby et al. 2017; Lee et al. 2022). In addition, wildlife may also act as a reservoir of ARB and reactor for the emergence of genes encoding antibiotic resistance (Guenther et al. 2011; Larsen et al. 2022). Wildlife has also been described as a source of pathogen and ARB introduction into the food chain (Greig et al. 2015), and extended‐spectrum beta‐lactamase (ESBL)‐producing *E. coli* carriage in the human population has partly been attributed to environmental sources, including wildlife, although data were lacking from non‐avian sources (Mughini‐Gras et al. 2019). Furthermore, it has been suggested that transmission of antibiotic resistance determinants could occur through the trophic chain in the environment (Jobbins and Alexander 2015). Despite this, information on occurrence of antibiotic‐resistant bacteria in top predators and opportunistic carnivores, which could potentially accumulate ARB originating from prey and carcasses, is scarce (Radhouani et al. 2014; Jobbins and Alexander 2015; Ramey and Ahlstrom 2020).

The need to integrate wildlife screening in surveillance programs for antibiotic resistance has been previously highlighted (Saied 2024). However, establishing such programs can be cumbersome and costly; therefore, it has been proposed to utilise animals already included in ongoing wildlife monitoring programs (Doyle et al. 2025). The use of wildlife as sentinels for ARB in the environment has been demonstrated in studies focusing on wild birds (Wyrsch et al. 2022; Woksepp et al. 2023; Nesporova et al. 2024). In contrast, research on terrestrial animals that interact with human populations in different ways remains limited.

In Sweden, there are several monitoring programs established for wildlife disease and health (SVA 2023). In addition, the Swedish Hunting Act stipulates that wildlife belonging to endangered, or otherwise valuable species, if found dead or killed, should be submitted to the State (Jaktlag 1987: 259). What is considered ‘state‐owned wildlife’ currently includes, among others, bears, wolves, eagles, and otters. The species stated in the ordinance should be submitted to either the Swedish Museum of Natural History (SMNH) or the Swedish Veterinary Agency (SVA). Moreover, the public is encouraged to report and submit wildlife found dead or diseased and euthanized to SVA. These established programs and submissions offer access and a cost‐efficient possibility to collect wildlife samples to be included in a monitoring program for ARB.

Besides having access to samples, there is a need for a suitable indicator to be used for ARB monitoring. *E. coli* has been proposed as an indicator for environmental surveillance of antibiotic resistance, and it is used in human and livestock monitoring programs (Anjum et al. 2021). ESBL‐producing *E. coli* is also used in the World Health Organization (WHO) tricycle protocol for multi‐sectorial surveillance on antibiotic resistance (WHO 2021). Furthermore, *E. coli* is present in most terrestrial animal species, and ESBL‐ and carbapenemase‐producing *E. coli* (CPEc) have been isolated from wildlife (Guenther et al. 2011; Dolejska and Literak 2019; Lagerstrom and Hadly 2021). Understanding genetic diversity and antibiotic resistance profiles in *E. coli* populations isolated from wildlife could therefore provide valuable information needed to understand the ARB transmission dynamics in the environment‐wildlife‐livestock‐human interface.

In the current study, we evaluated the occurrence of ESBL‐ and plasmid‐mediated AmpC‐producing (pAmpC) isolates, CPEc and indicator *E. coli* in samples collected in conjunction with ongoing wildlife monitoring programs in Sweden, and phenotypically and genotypically characterized identified isolates. The samples were collected in conjunction with ongoing monitoring programs for wildlife health and diseases at SVA and SMNH. The targeted animals in this study included brown bears ( *Ursus arctos* ), white‐tailed and golden eagles ( *Haliaeetus albicilla* and *Aquila chrysaetos* ), Eurasian wolves ( *Canis lupus lupus* ), red foxes ( *Vulpes vulpes* ), European and mountain hares ( *Lepus europaeus* and *Lepus timidus* ), and Eurasian otters ( *Lutra lutra* ). The aim of the study was to determine the occurrence of ARB in the selected wildlife species utilising a similar methodology as the one used for antibiotic resistance monitoring in surveillance programs for food producing animals in Sweden (Folkhälsomyndigheten and SVA 2023). In addition, the study also aimed to determine if ongoing surveillance programs could be utilised in future antibiotic resistance surveillance programs on wildlife.

## 2 Materials and Methods

### 2.1 Sample Collection

A total of 358 faecal samples from six wild animal species were collected between February 2020 and December 2023. Samples from brown bears ( *Ursus arctos* ), white‐tailed eagles ( *Haliaeetus albicilla* ), golden eagles ( *Aquila chrysaetos* ), Eurasian wolves ( *Canis lupus lupus* ), red foxes ( *Vulpes vulpes* ), European hares ( *Lepus europaeus* ) and mountain hares ( *Lepus timidus* ) were collected by the pathology department at SVA during necropsy (Table 1). Samples from Eurasian otters ( *Lutra lutra* ) were collected by the SMNH (Table 1). Brown bears, eagles, wolves and otters were submitted to SVA and SMNH for necropsy, as part of ‘state owned wildlife’. Bears and wolves mainly originated from protective hunting (i.e., animals hunted to protect livestock), while the majority of foxes were hunted foxes submitted to SVA as part of a specific monitoring program for fox tapeworm (*Echinococcus multilocularis*) (SVA 2022). The included hares were submitted to SVA for general health surveillance of fallen wildlife (SVA 2023). The geographical origin of each animal was registered upon arrival of the carcasses. Most carcasses were stored at −20°C and thawed prior to necropsy. State of decomposition varied, but severely decomposed carcasses were not sampled.

**TABLE 1:** Number of samples cultured, indicator *E. coli* isolates collected, ESBL‐ and pAmpC‐producing, and carbapenemase‐producing *E. coli* isolates found per animal species.

Source	Number of samples	Number of samples positive for *E. coli*	Number of indicator *E. coli* isolates resistant to at least one antibiotic	Number of ESBL‐ and pAmpC‐producing *E. coli* isolates	Number of carbapenemase‐producing *E. coli* isolates
Brown bear	108	86 (80%)	3	0	0
White‐tailed eagle	31	18 (58%)	2	0	0
Golden eagle	4	2 (50%)	1	0	0
Eurasian wolf	20	17 (85%)	0	0	0
Red fox	84	62 (74%)	1	2	0
European hare	49	18 (37%)	0	0	0
Mountain hare	3	2 (67%)	0	0	0
Eurasian otter	59	8 (14%)	0	0	0

FLOQSwabs eSwabs (Copan, Italy) were used to collect samples from the bowel, anal canal or the cloaca and stored at −80°C after adding 20% glycerol.

Data on human population density per county in Sweden for 2022 was obtained from the Statistics Sweden database, accessed June 2025 (https://www.statistikdatabasen.scb.se/).

### 2.2 Cultivation of *E. coli* and ESBL‐, pAmpC and Carbapenemase‐Producing *E. coli*

Samples from brown bears and eagles were thawed, and 10 μL medium was transferred to 2 mL of Brain Heart Infusion (BHI) broth (Oxoid Ltd., United Kingdom) supplemented with vancomycin 8 mg/L. Samples from wolves, red foxes, hares, and otters were thawed, and 10 μL of the swab medium was transferred to 5 mL of buffer peptone water (Millipore, France). All samples were incubated at 37°C for 20 ± 2 h under aerobic conditions. After pre‐enrichment, 10 μL of the broth were inoculated on CHROMagar Orientation, CHROMagar C3GR, and CHROMagar mSuperCarba agar (CHROMagar, France), and incubated at 37°C for 20 ± 2 h. *Escherichia coli* ATCC25922, *Klebsiella pneumoniae* CCUG45421, and *Escherichia coli* NCTC13476 were used as quality control strains.

From each sample and agar, one colony with a morphology corresponding to *E. coli* , according to the manufacturer's instructions, was selected, and species confirmation was performed using Matrix Assisted Laser Desorption/Ionization Time Of Flight (MALDI‐TOF) microflex instrument (Bruker, Germany), using the MBT Compass Library (Bruker, version 2022, RUO). Identification was based on the manufacturer's recommended score thresholds ≥ 2.0 for species identification.

### 2.3 Antimicrobial Susceptibility Testing

Antimicrobial susceptibility testing was performed using disk diffusion on Mueller‐Hinton agar (in‐house), following the protocol described by the European Committee on Antimicrobial Susceptibility Testing (EUCAST), and using clinical breakpoints for *Enterobacterales* from EUCAST v. 11. Thirteen antibiotics were tested: Ampicillin (10 μg), cefadroxil (30 μg), chloramphenicol (30 μg), ciprofloxacin (5 μg), gentamycin (10 μg), mecillinam (10 μg), meropenem (10 μg), nalidixic acid (30 μg), nitrofurantoin (100 μg), piperacillin/tazobactam (30/6 μg), trimethoprim/sulfamethoxazole (1.25/23.75 μg), tetracycline (30 μg), and trimethoprim (5 μg) (Oxoid Ltd., United Kingdom).

Isolates from C3GR agar were also tested using double disk synergy testing (EUCAST), using amoxicillin/clavulanic acid (20/10 μg) in combination with cefepime (30 μg), cefoxitin (30 μg), ceftazidime (10 μg), and cefotaxime (5 μg) (Oxoid Ltd., United Kingdom).

All isolates presenting phenotypic resistance to ≥ 1 antibiotic, together with a randomly selected subset of sensitive isolates (up to 20 per animal species) were selected for sequencing.

### 2.4 Whole Genome Sequencing and Sequence Analysis

Isolates were sequenced at the Science for Life Laboratory Clinical Genomics Facility (Solna, Sweden) on a NovaSeq X platform (Illumina) as 2 × 150 bp paired‐end.

The raw reads were downsampled to 120× before trimming Illumina adapters and low‐quality regions using Trimmomatic 0.39 (Bolger et al. 2014). Quality control of the trimmed sequences was performed using FastQC 0.12.0 (Andrews 2010). The trimmed and paired sequences were assembled using SPAdes Genome Assembler v. 4.0.0 (Bankevich et al. 2012), specifying the isolate assembly mode.

Screening for antibiotic resistance genes was performed using staramr (Bharat et al. 2022), using the ResFinder database (September 2024), using an identity threshold of > 90% and > 95% coverage. Identification of point mutations conferring antibiotic resistance was performed using AMRFinder (accessed June 2025) (Feldgarden et al. 2019). Screening of virulence factors was performed using the VirulenceFinder 2.0 database (Joensen et al. 2014) (accessed June 2025), specifying *E. coli* as organism, with an identity threshold of 95% and 100% coverage.

The raw reads were uploaded to EnteroBase (accessed June 2025) (https://enterobase.warwick.ac.uk/species/index/ecoli) for species confirmation, phylogroup determination, Achtman 7 gene multi‐locus sequence typing (MLST), core‐genome multi‐locus sequence typing (cgMLST), serotype identification, and *fimH‐typing* (Zhou et al. 2020).

Average nucleotide identity (ANI) for isolates belonging to *Escherichia* cryptic clade V was calculated using JSpeciesWS (https://jspecies.ribohost.com/jspeciesws/) (Richter et al. 2016), using *Escherichia marmotae* YF8 (GCA_029962465.1) as a reference genome.

Any identified ESBL and pAmpC‐producing isolate and indicator *E. coli* isolates carrying antibiotic resistance genes, isolates presenting *stx* genes, and isolates belonging to extraintestinal pathogenic *E. coli* (ExPEC) STs ST10, ST58, ST131, and ST648 were further investigated for genomic relatedness with other genomes in EnteroBase (June 2025), using the hierarchical clustering (HierCC) designations (Zhou et al. 2021). Minimum spanning trees were constructed based on cgMLST V1 and HierCC V1 in EnteroBase using GrapeTree with the NINJA NJ algorithm.

## 3 Results

### 3.1 Low Occurrence of ESBL and pAmpC‐Producing *E. coli* and no Carbapenemase‐Producing *E. coli* in Swedish Wildlife

Of the 358 samples, two samples were positive for ESBL and pAmpC‐producing *E. coli* , both from red foxes, and no CPEc could be detected (Table 1). The isolates were confirmed as ESBL‐producing, carrying *bla*_CTX‐M‐15_ and *bla*_OXA‐1_, and pAmpC‐producing, carrying *bla*_DHA‐1_. The isolates belonged to different phylogroups, STs and serotypes (Table 2), and were determined to be multidrug resistant (MDR) (resistant ≥ 3 antibiotic classes).

**TABLE 2:** Additional phenotypic antibiotic resistance and genetic determinants for ESBL‐ and pAmpC‐producing *E. coli* isolates.

Source	Isolate ID	Phylogroup	ST	Serotype	*fimH* type	Phenotypic resistance	Genetic determinants
Red fox	R7b	B2	ST1193	O75:H5	*fimH64*	Gentamycin	*aac(3)‐IIa*
							*aac(6′)‐Ib‐cr*
							*aph(3″)‐Ib*
							*aph(6)‐Id*
						Trimethoprim	*dfrA17*
						Sulfisoxazole	*sul2*
						Tetracycline	*tet(B)*
						Ciprofloxacin	*gyrA* ^#^ (D87N)
							*gyrA* ^#^ (S83L)
							*parC* ^#^ (S80I)
							*parE* ^#^ (L416F)
Red fox	R8b	B1	ST200	O175:H31	*fimH31*	Azithromycin	*mph(A)*
						Ciprofloxacin	*qnrB4*
						Sulfisoxazole	*sul1*
						Trimethoprim	*dfrA17*

The ESBL‐producing isolate (R7b) carried several virulence‐associated genes (VAGs), and the pAmpC‐producing isolate (R8b) carried several VAGs linked with an *E. coli* enteroaggregative pathotype (EAEC) (Supporting Information 1).

Based on cgMLST, the ESBL‐producing isolate (R7b, ST1193) clustered with several human genomes, including some from blood and urine, with no more than 10 allele differences (HC10 4073) (Figure S1). The pAmpC‐producing isolate (R8b, ST200) clustered together with genomes originating from human samples with links no more than 50 alleles apart (HC50 21668) (Figure S2).

### 3.2 Low Occurrence of Antibiotic Resistance in Indicator *E. coli* Isolated From Wildlife in Sweden

In total, 213 *E. coli* isolates were collected from 358 (59.5%) faecal samples (Table 1). Of the identified *E. coli* isolates, 206 (96.7%) were fully susceptible (Table 1) and 7 isolates (2.3%) were resistant to ≥ 1 antibiotic (Tables 1 and 3). Two of the isolates presented phenotypic resistance against ampicillin but were not positive for any acquired antibiotic resistance genes or chromosomal mutations. None of the isolates originating from wolves or hares carried acquired antibiotic resistance genes. Zone diameters and cut‐off values for indicator *E. coli* isolates can be found in Supporting Information 3.

**TABLE 3:** Genotypic characterization and correspondence between reported phenotypic antibiotic resistance and genetic determinants for indicator *E. coli* .

Source	Isolate ID	Phylogroup	ST	Serotype	Phenotypic resistance	Genetic determinants
Brown bear	1031a1Bj	C	ST88	O8:H4	Ampicillin	*bla*_TEM‐1B_
					Tetracycline	*tet(A)*
Brown bear	1025a1Bj	F	ST1276	O8:H51	Trimethoprim	*dfrA5*
Brown bear	1341a1	B1	ST2602	O8:H16	Ampicillin	—
White‐tailed eagle	1095a2	C	ST88	O8:H4	Ampicillin	*bla*_TEM‐1B_
					Tetracycline	*tet(A)*
White‐tailed eagle	578a	B1	ST4198	O75:H38	Ampicillin	—
					Trimethoprim	*dfrA5*
*Golden eagle*	321a2	B1	ST162	B14/O32:H19	Ciprofloxacin	*gyrA* ^#^ D87N
						*gyrA* ^#^ S83L
						*parC* ^#^ S80I
Red fox	R6a	C	ST88	O8:H4	Gentamycin	*aph(3″)‐Ib*
						*aph(3′)‐Ia*
						*aph(6)‐Id*
					Ampicillin	*bla*_TEM‐1B_
					Trimethoprim	*dfrA5*
					Sulfisoxazole	*sul2*

Mutations in the *pmrB* gene corresponding to the amino acid switches E123D and Y358N, linked to colistin resistance, were found in 29 and 46 of the sequenced isolates.

### 3.3 Characterization of Antibiotic Susceptible Indicator *E. coli*

A total of 92 fully susceptible indicator *E. coli* isolates were randomly selected for sequencing. The isolates originated from brown bears (*n* = 17), eagles (*n* = 15), wolves (*n* = 15), foxes (*n* = 18), hares (*n* = 19), and otters (*n* = 8).

**FIGURE 1:**
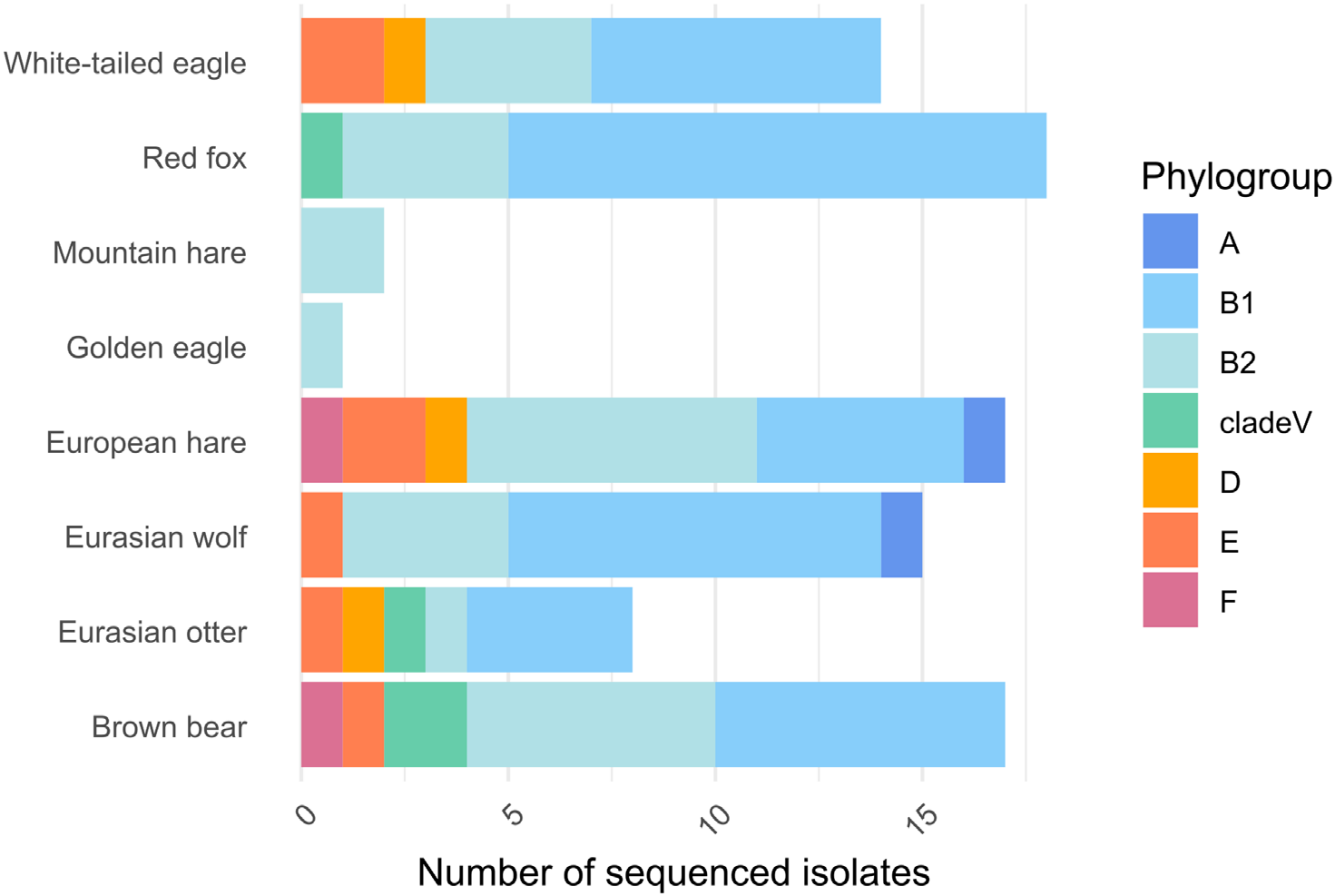
Phylogroup distribution of antibiotic susceptible indicator *E. coli* isolates by source.

The isolates were distributed among six phylogroups (Figure 1). The most common phylogroup was B1 (45 isolates; 48.9%), followed by B2 (29 isolates; 31.5%) and E (7 isolates; 7.6%). The remaining isolates were distributed between phylogroups A (2 isolates; 2.2%), D (3 isolates; 3.3%) and F (2 isolates; 2.2%). Four isolates belonged to *Escherichia* clade V (4.3%) and were determined to be *Escherichia marmotae* based on ANI.

MLST analysis revealed diversity among the isolates from all sources, distributed in 81 sequence types (STs). The most represented sequence types (ST) were ST88, ST131, ST446, and ST2521, with three isolates each (3%). Serotype identification and *fimH* typing also showed a high diversity among the isolates (Supporting Information 4).

Isolates belonging to ST10 and ST648 did not cluster with other isolates in EnteroBase with links no more than 100 alleles apart (under the HierCC HC100 designations), while the isolate belonging to ST58 (R25a) clustered with genomes originating from poultry, swine, cattle, and humans, with links no more than 100 alleles apart (HC100 25133). One isolate belonging to ST131 (V3a) clustered together with genomes originating from humans, with links no more than 50 alleles apart (HC50 7090); and clustered together with the other two ST131 isolates (H1537Ec and H4567Ec), and with genomes originating from human, environmental, and animal sources, with links no more than 100 alleles apart (HC100 58).

Isolates presenting antibiotic resistance genes and typed as ST88 (1031a1Bj and R6a) clustered together, and with other genomes from humans, domestic and wild animals with links no more than 100 alleles apart (HC100 30962) (Figure S3).

One isolate originating from an otter carried the gene *fosA7* that codes for resistance against fosfomycin (not tested during phenotypic screening).

### 3.4 Detection of Shiga‐Toxin Producing *E. coli* (STEC) Isolates

All indicator *E. coli* isolates carried virulence‐associated genes (VAGs), with varying numbers and functions. Isolates classified into phylogroup A and clade V (*E. marmotae)* generally presented a low number of VAGs, while isolates classified into B2 and C presented a high number of VAGs. For details, see Supporting Information 4.

Two indicator *E. coli* isolates carried genes encoding shiga toxins (Table 4). One of the isolates (1010a1Bj) carried additional VAGs associated with adhesion and iron uptake; and the other isolate (V14a) carried additional VAGs associated with adherence, enterohaemolysin production, iron uptake, and complement resistance (Supporting Information 4). None of the isolates carried *eae* genes or genes or mutations encoding antibiotic resistance.

**TABLE 4:** Genetic determinants of STEC isolates.

Source	Isolate ID	Phylogroup	ST	Serotype	*fimH* type	*stx* variant
Brown bear	1010a1Bj	B1	ST17448	O‐:H49	*fimH1084*	*stx1a*
Eurasian wolf	V14a	B1	ST26	O‐:H8	*fimH31*	*stx2b*

Based on cgMLST, the *stx1a*‐positive isolate (1010a1Bj, ST17448) clustered together with human genomes with links no more than 50 alleles apart (HC50 1865); and the isolate carrying *stx2b* (V14a, ST26) was clustered with genomes from deer, with links no more than 50 alleles apart (HC50 29641).

### 3.5 Origin of the Samples and Correlation to Human Population Density

**FIGURE 2:**
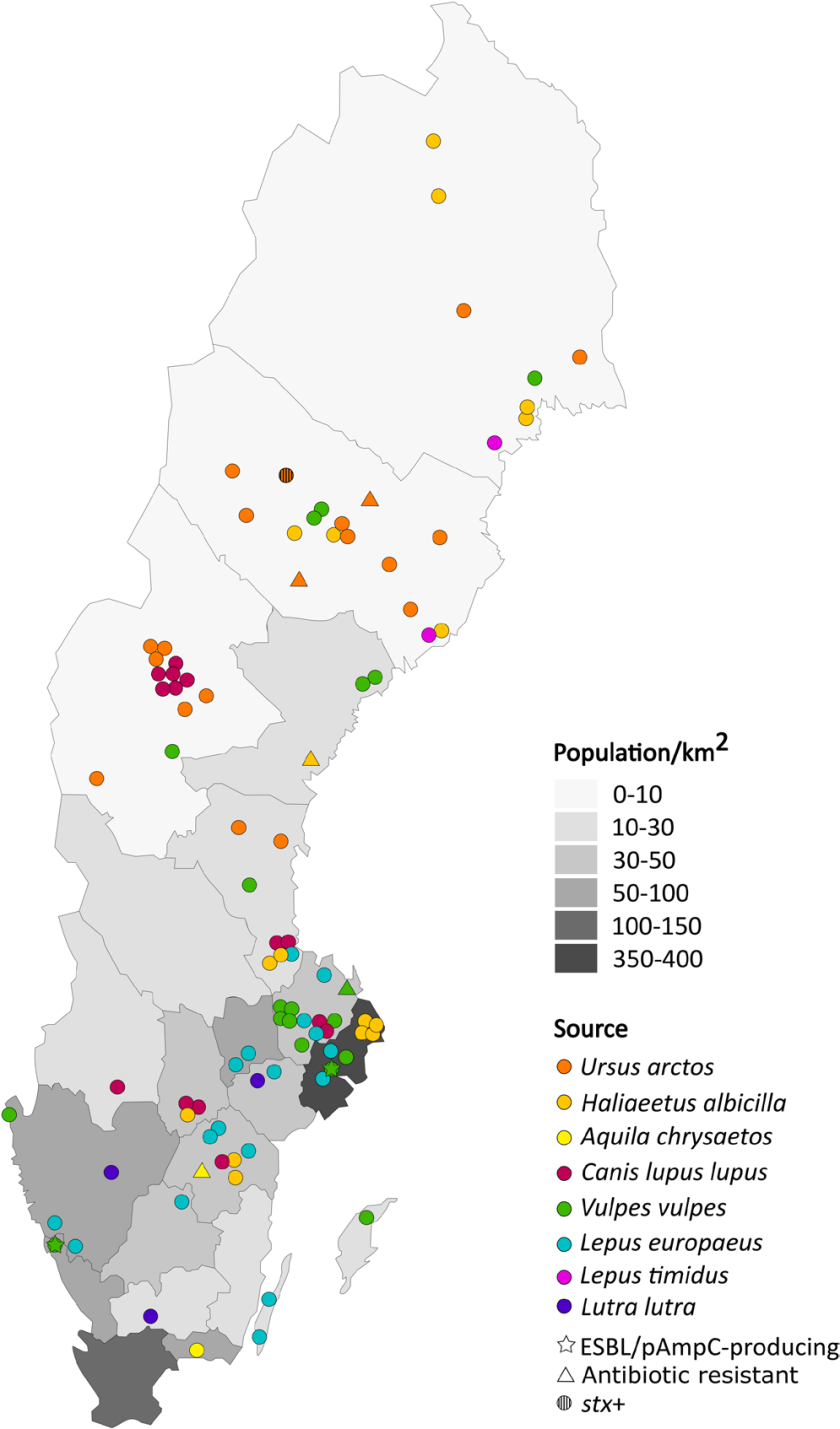
Sample origin of the sequenced isolates and human population density per county. Colouring is based on animal species. ESBL‐, pAmpC‐producing, antibiotic‐resistant isolates and *stx* + isolates are marked according to the legend.

Most of the sequenced strains were isolated from samples originating from areas with low population densities (Figure 2). Information on the municipality was not available for nine of the bear samples (represented in the county they originated from). Information on the origin of the samples was not available for five of the otter samples and one of the wolf samples (not represented in the figure). The ESBL‐producing isolate originated from a sample from Stockholm and the pAmpC‐producing isolate originated from a sample from Kungsbacka municipality in Halland County. Information on the origin of all samples positive for indicator *E. coli* can be found in Supporting Information 5.

## 4 Discussion

In the current study, no CPEc could be detected, but two foxes carried ESBL‐ and pAmpC‐producing *E. coli* . These results are in stark contrast with previous studies in Sweden focusing on wildlife and environment, as showcased by studies in mallards, where the detection rate of ESBL‐producing *Enterobacteriaceae* was 47% (Hessman et al. 2018); gulls, where up to 17% of the samples carried ESBL‐producing *E. coli* and some samples were positive for CPEc (Wallensten et al. 2011; Atterby et al. 2017; Woksepp et al. 2023); and surface waters, where 27/98 samples were positive for the detection of ESBL/pAmpC *E. coli* (Egervärn et al. 2017). In addition, the carriage of ESBL/pAmpC *E. coli* is lower than reported in humans in Sweden (4.7%), and lower or at a similar level as those reported in healthy livestock (0%–3%) (Ny et al. 2017; Folkhälsomyndigheten and SVA 2023, 2024). However, the carriage of ESBL‐producing *E. coli* is similar to that previously reported in Swedish corvids (2%), where the authors suggested that the bird carrying the ESBL‐producing strain likely acquired it from the environment, and could be related to a high human and livestock population density in the area (Söderlund et al. 2019).

In foxes, the occurrence of ESBL/pAmpC *E. coli* , corresponding to 2/62 isolates (3.2%), is comparable to that reported in Norway (3.4%) (Mo et al. 2018), but lower compared to Northern Ireland (11.5%) and Poland (10.5%) (O'Hagan et al. 2021; Osińska et al. 2022). Both the ESBL and pAmpC‐producing isolates in our study originated from fox samples from areas with a high human population density, indicating that proximity to anthropogenic settings could have an impact on the carriage of antibiotic resistance in this species. Red foxes constitute a generalist species, demonstrating high adaptability to urban environments (Walton et al. 2017), and exploiting anthropogenic food sources (Fletcher et al. 2025). Previous studies have suggested that increasing synanthropization may drive higher resistance levels in foxes (Osińska et al. 2022), supporting the hypothesis that anthropogenic contact influences antibiotic resistance occurrence in wildlife. A similar association has been reported in Norway (Mo et al. 2018), where all ESBL and pAmpC‐producing isolates originated from samples from urban areas. Genotypic characterization of the ESBL‐ and pAmpC‐producing isolates in our study further supports the hypothesis that anthropogenic contact plays a role in carriage of ARB in foxes. The ESBL‐producing isolate belonged to ST1193 (O75:H5 *fimH64*), and carried a large set of VAGs (Supporting Information 1) linked with ExPEC pathogenicity (Bélanger et al. 2011). *E. coli* ST1193‐O75:H5*‐H64* has been described as a MDR emerging pandemic ExPEC clone (Johnson et al. 2019; Pitout et al. 2022). This isolate carried the *bla*_CTX‐M‐15_ gene, a globally disseminated ESBL gene previously found in clinical and environmental isolates (Bonnevie et al. 2025; Edman‐Wallér et al. 2025; Elmarghani et al. 2025). The pAmpC‐producing isolate belonged to ST200, was MDR and carried multiple VAGs linked with an EAEC pathotype (Van Nederveen and Melton‐Celsa 2025). ST200 isolates have been previously linked with EAEC pathogenicity in Spain and India (Llorente et al. 2023; Modgil et al. 2024). In addition, both isolates clustered with other isolates of human origin (Figures S1 and S2).

Resistant indicator *E. coli* from the animals in our study was uncommon, with only resistant isolates verified from bears (*n* = 2), eagles (*n* = 2), foxes (*n* = 1) and otters (*n* = 1) (Table 3). This is in contrast to reports from other European countries (Pinto et al. 2010; Guenther et al. 2012; Radhouani et al. 2012; Simões et al. 2012; Mo et al. 2018). Nevertheless, the prevalence of antibiotic resistance in Sweden is low compared to other countries (EFSA and ECDC 2024), and thus a lower carriage rate of resistant *E. coli* compared to other countries could be expected. In addition, a large proportion of the animals sampled in our study originated from areas with a low human population density. Thus, limited contact between wildlife and urban areas could also explain the low occurrence. Similar results have been observed in areas with limited anthropogenic influence in Europe (Literak et al. 2010; Kozoderović et al. 2021; Homeier‐Bachmann et al. 2022; Mengistu et al. 2022). Differences in diet have been observed in wolves and otters according to their distribution in Northern versus Southern Europe (Torres and Fonseca 2016; Ståhlberg et al. 2017), and could therefore have an impact on the carriage of antibiotic resistance in wildlife in different regions. However, comparisons with previous studies should be made carefully, as there are differences regarding the methods employed and the number of samples examined per animal species. Furthermore, although selection of one indicator isolate per sample is in line with standardised screening in surveillance programs, this approach might underestimate the true within‐sample diversity. Nevertheless, the low level of resistance in otters and eagles was surprising, as otters constitute a generalist species and top predator in freshwater environments (Loy et al. 2022); and eagles represent apex predators and scavengers. The low isolation rate of *E. coli* might also have influenced these results, which is likely due to limitations during sampling, as carcasses were frozen prior to sampling and collected samples were frozen prior to analysis, which is known to affect the recovery rate of *E. coli* (Masters et al. 2015); and the decomposition state of carcasses upon thawing varied. In eagles, samples were taken using cloacal swabs, which are known to be less representative of the gut microbiota than faecal swabs (Videvall et al. 2018).

From the seven isolates resistant to > 1 antibiotic, three belonged to phylogroup B1, and three were classified into phylogroup C and ST88. The three ST88 isolates carried ARGs, several genes associated with ExPEC virulence and antibiotic‐resistance genes, and clustered with isolates from humans and diverse animal sources in EnteroBase (Figure S3). Strains belonging to phylogroup C are usually associated with a lower number of human ExPEC infections compared to strains from phylogroups B2 and D, but previous studies have suggested this phylogroup as a potential reservoir of antibiotic resistance (Massot et al. 2016; Wang et al. 2023). One of the isolates belonged to phylogroup F and carried a resistance gene against trimethoprim and several VAGs. While less common, extraintestinal infections attributed to strains that belong to phylogroup F have been reported (Lau et al. 2008; Guo et al. 2015; Wang et al. 2023). A large proportion of the indicator isolates presented mutations in the *pmrB* gene, previously linked with colistin resistance (Delannoy et al. 2017; Luo et al. 2017; Furlan et al. 2022). However, colistin susceptibility was not evaluated during phenotypic screening, as it has previously been established that these mutations likely are not an indication of true resistance but rather related to phylogroup distribution (Butters et al. 2024).

To fully comprehend the potential anthropogenic effect, and to evaluate the potential risk for human and domestic animal health, it is essential to understand the *E. coli* population structure in wildlife, as the genetic background of different strains plays an important role in the carriage of antibiotic resistance and virulence‐associated genes (Clermont et al. 2019; Denamur et al. 2021; Petitjean et al. 2021). Therefore, we sequenced a random selection of fully susceptible indicator *E. coli* isolates. These isolates showed high genetic diversity, with no clear differences in phylogroup or ST distribution between animal species. The most represented phylogroup was B1, which is the most commonly described phylogroup in wild animals (Gordon and Cowling 2003; Tenaillon et al. 2010; Lagerstrom and Hadly 2023). Phylogroup B2, that accounts for a large percentage of human ExPEC infections (Halaji et al. 2022; Micenková et al. 2016), was also identified in all animals, and generally carried a high number of VAGs. However, the relatively large VAG carriage within this phylogroup is likely linked with commensalism (Le Gall et al. 2007; Köhler and Dobrindt 2011). Among the B2 isolates, two belonged to ST131, but only one clustered with isolates from humans in EnteroBase, including some from blood and urine. This isolate carried several VAGs, indicating potential anthropogenic association and pathogenicity. However, it did not carry the *fimH30 or fimH41* alleles associated with epidemic ESBL‐producing *E. coli* ST131 (Johnson et al. 2013; Price et al. 2013). Four of the isolates were re‐identified as *Escherichia marmotae* and carried the *astA* gene, that encode heat‐stable enterotoxin EAST1 (Ménard and Dubreuil 2002). *E. marmotae* has recently gained increased attention in clinics but was originally described in high occurrence in wild marmot ( *Marmota himalayana* ) (Liu et al. 2015; Sivertsen et al. 2022). However, our isolates lacked other genes associated with pathogenicity, and carried less VAGs than the other phylogroups, suggesting limited pathogenic potential.

Among the susceptible isolates, two were identified as shiga‐toxin producing *E. coli* , and originated from a bear and a wolf sample. To the best of our knowledge, this is the first report of STEC isolated from brown bears and Eurasian wolves. A previous study recovered an *stx*_*1*_‐positive STEC from a wild wolf (*C. lupus italicus*) in Italy, but this isolate was not further characterized (Bertelloni et al. 2024). STECs are traditionally considered to have a major reservoir in domestic ruminants, but can also be found in a wide range of wild birds and mammals, particularly deer (Espinosa et al. 2018). Comparison of the isolates with genomes in Enterobase revealed that the wolf isolate clustered with deer isolates from multiple countries, indicating that predation or scavenging on deer could constitute a route of STEC exposure for carnivores. In general, STEC isolates carrying *stx*_*1a*_ or *stx*_*2b*_ cause a significant proportion of human STEC cases, but are rarely associated with major outbreaks or the most severe cases of disease, and are regarded as lower risk for human health (FAO and WHO 2018). In Sweden, ST26 caused 19 domestic cases of human STEC infection between 2023 and 2025, while there are no reported cases caused by STEC ST17448 during the same period (Lina Thebo, The Public Health Agency of Sweden, personal communication).

Overall, the results of the current study demonstrate a low occurrence of antibiotic resistance in Swedish wildlife, reflecting limited anthropogenic impact, and the generally low occurrence of ARB in livestock and humans in Sweden. The species included in our study, especially foxes, could serve as potential sentinel species, as these animals are synanthropic to varying degrees and have previously been shown to carry antibiotic‐resistant bacteria in other European countries. Even though the occurrence of antibiotic resistance in otters and eagles in our study was low, these species could potentially be used as sentinels, as they are closely linked to aquatic ecosystems, exhibit synanthropic habits, and are widely distributed throughout the country. Despite some limitations, our study illustrates that ongoing wildlife monitoring programs could be used as a cost‐efficient approach for antibiotic resistance surveillance in wildlife and the environment. Our results demonstrate that future surveillance programs should take careful consideration when choosing wildlife species composition, as distribution, habitat, and behaviour may heavily influence the results.

## Author Contributions

**Robert Söderlund:** conceptualization, methodology, validation, writing – review and editing, supervision. **María Rincón‐Gracia:** investigation, writing – original draft, writing – review and editing, visualization, formal analysis, data curation, methodology, validation. **Aleksija Neimanis:** methodology, investigation, resources, writing – review and editing. **Jonas Bonnedahl:** conceptualization, methodology, validation, funding acquisition, writing – review and editing, writing – original draft, supervision, project administration. **Hugo Uddströmer:** investigation, writing – review and editing. **Thomas Rosendal:** formal analysis, conceptualization, supervision, writing – review and editing. **David Winlund:** investigation, writing – review and editing. **Hanna Woksepp:** validation, investigation, resources, writing – review and editing, supervision. **Josef D. Järhult:** conceptualization, resources, writing – review and editing, funding acquisition. **Henrik Uhlhorn:** methodology, investigation, resources, writing – review and editing. **Stefan Börjesson:** conceptualization, funding acquisition, writing – original draft, writing – review and editing, supervision, project administration, methodology, validation.

## Funding

This work was supported by the Horizon 2020 Framework Programme (869178‐AquaticPollutants), Svenska Forskningsrådet Formas (FR‐2021/0001), and Vetenskapsrådet (2020‐05697).

## Ethics Statement

The study does not include human subjects, and ethical approval according to national legislation (SJVFS 2015: 38) was not required, as no animals were killed for the purpose of this study.

## Conflicts of Interest

The authors declare no conflicts of interest.

## Supporting information


**Table S1:** Virulence associated genes for ESBL‐ and pAmpC‐producing isolates.

**Figure S1:** Minimum spanning tree based on isolates clustered in HC10 4073. Colorednodes are based on source type. The number of isolates per source type is shown in parentheses [*n*]. The ESBL‐producing isolate (R7b) is represented as Other Mammal.
**Figure S2:** Minimum spanning tree based on isolates clustered in HC50 21668. Coloured nodes are based on source type. The number of isolates per source type is shown in parentheses [*n*]. The pAmpC‐producing isolate (R8b) is represented as Other Mammal.
**Figure S3:** Minimum spanning tree based on isolates clustered in HC100 30962. Coloured nodes are based on source type. The number of isolates per source type is shown in parentheses [*n*]. Isolates belonging to ST88 (1031a1Bj and R6a) are represented as Other Mammal.

**Table S2:** Zone diameters and cut‐off values for indicator *E. coli* isolates.

**Table S3:** Atchmant 7 gene MLST, phylogroup distribution, serotype, *fimH* type and virulence associated genes of indicator *E. coli* isolates.

**Figure S4:** Sample origin of bear samples and human population density per county. Information on the geographical origin of two samples was not available (not represented in the figure). Information on municipality was not available for nine of the bear samples (represented in the county they originated from).
**Figure S5:** Sample origin of eagle samples and population density per county.
**Figure S6:** Geographical origin of wolf samples and human population density per county. Information on geographical origin was not available for one of the samples (not represented in the figure).
**Figure S7:** Geographical origin of fox samples and human population density per county. ESBL‐, pAmpC‐producing and antibiotic‐resistant isolates are marked according to the legend.
**Figure S8:** Geographical origin of hare samples and human population density per county.
**Figure S9:** Geographical origin of otter samples and human population density per county. Information on geographical origin was not available for five of the samples (not represented in the figure).

## Data Availability

The raw sequencing data for this study has been deposited in the European Nucleotide Archive (ENA) at EMBL‐EBI under accession number PRJEB108103.

## References

[emi470404-bib-0001] Ahlstrom, C. A. , M. L. Van Toor , H. Woksepp , et al. 2021. “Evidence for Continental‐Scale Dispersal of Antimicrobial Resistant Bacteria by Landfill‐Foraging Gulls.” Science of the Total Environment 764: 144551. 10.1016/j.scitotenv.2020.144551. 33385653

[emi470404-bib-0002] Allen, H. K. , J. Donato , H. H. Wang , K. A. Cloud‐Hansen , J. Davies , and J. Handelsman . 2010. “Call of the Wild: Antibiotic Resistance Genes in Natural Environments.” Nature Reviews Microbiology 8, no. 4: 251–259. 10.1038/nrmicro2312. 20190823

[emi470404-bib-0003] Andrews, S. 2010. “A Quality Control Tool for High Throughput Sequence Data.” https://www.bioinformatics.babraham.ac.uk/projects/fastqc/.

[emi470404-bib-0004] Anjum, M. F. , H. Schmitt , S. Börjesson , et al. 2021. “The Potential of Using *E. coli* as an Indicator for the Surveillance of Antimicrobial Resistance (AMR) in the Environment.” Current Opinion in Microbiology 64: 152–158. 10.1016/j.mib.2021.09.011. 34739920

[emi470404-bib-0005] Atterby, C. , S. Börjesson , S. Ny , J. D. Järhult , S. Byfors , and J. Bonnedahl . 2017. “ESBL‐Producing *Escherichia coli* in Swedish Gulls—A Case of Environmental Pollution From Humans?” PLoS One 12, no. 12: e0190380. 10.1371/journal.pone.0190380. 29284053 PMC5746268

[emi470404-bib-0006] Bankevich, A. , S. Nurk , D. Antipov , et al. 2012. “SPAdes: A New Genome Assembly Algorithm and Its Applications to Single‐Cell Sequencing.” Journal of Computational Biology 19, no. 5: 455–477. 10.1089/cmb.2012.0021. 22506599 PMC3342519

[emi470404-bib-0007] Bélanger, L. , A. Garenaux , J. Harel , M. Boulianne , E. Nadeau , and C. M. Dozois . 2011. “ *Escherichia coli* From Animal Reservoirs as a Potential Source of Human Extraintestinal Pathogenic *E. coli* .” FEMS Immunology and Medical Microbiology 62, no. 1: 1–10. 10.1111/j.1574-695X.2011.00797.x. 21362060

[emi470404-bib-0008] Bertelloni, F. , G. Cagnoli , and V. V. Ebani . 2024. “Survey on the Occurrence of Zoonotic Bacterial Pathogens in the Feces of Wolves ( *Canis lupus* Italicus) Collected in a Protected Area in Central Italy.” Microorganisms 12, no. 11: 2367. 10.3390/microorganisms12112367. 39597755 PMC11596315

[emi470404-bib-0009] Bharat, A. , A. Petkau , B. P. Avery , et al. 2022. “Correlation Between Phenotypic and in Silico Detection of Antimicrobial Resistance in *Salmonella enterica* in Canada Using Staramr.” Microorganisms 10, no. 2: 292. 10.3390/microorganisms10020292. 35208747 PMC8875511

[emi470404-bib-0010] Bolger, A. M. , M. Lohse , and B. Usadel . 2014. “Trimmomatic: A Flexible Trimmer for Illumina Sequence Data.” Bioinformatics 30, no. 15: 2114–2120. 10.1093/bioinformatics/btu170. 24695404 PMC4103590

[emi470404-bib-0011] Bonnevie, A. , M. Myrenås , and O. Nilsson . 2025. “ESBL‐ and pAmpC‐Producing Enterobacterales From Swedish Dogs and Cats 2017–2021: A Retrospective Study.” Acta Veterinaria Scandinavica 67, no. 1: 2. 10.1186/s13028-024-00786-2. 39762972 PMC11702106

[emi470404-bib-0012] Butters, A. , J. Jovel , S. Gow , K. Liljebjelke , C. Waldner , and S. L. Checkley . 2024. “PmrB Y358N, E123D Amino Acid Substitutions Are Not Associated With Colistin Resistance but With Phylogeny in *Escherichia coli* .” Microbiology Spectrum 12, no. 10: e00532‐24. 10.1128/spectrum.00532-24. 39162501 PMC11451601

[emi470404-bib-0013] Clermont, O. , O. V. A. Dixit , B. Vangchhia , et al. 2019. “Characterization and Rapid Identification of Phylogroup G in *Escherichia coli* , a Lineage With High Virulence and Antibiotic Resistance Potential.” Environmental Microbiology 21, no. 8: 3107–3117. 10.1111/1462-2920.14713. 31188527

[emi470404-bib-0014] Delannoy, S. , L. Le Devendec , E. Jouy , P. Fach , D. Drider , and I. Kempf . 2017. “Characterization of Colistin‐Resistant *Escherichia coli* Isolated From Diseased Pigs in France.” Frontiers in Microbiology 8: 2278. 10.3389/fmicb.2017.02278. 29209292 PMC5702452

[emi470404-bib-0015] Denamur, E. , O. Clermont , S. Bonacorsi , and D. Gordon . 2021. “The Population Genetics of Pathogenic *Escherichia coli* .” Nature Reviews Microbiology 19, no. 1: 37–54. 10.1038/s41579-020-0416-x. 32826992

[emi470404-bib-0016] Dolejska, M. , and I. Literak . 2019. “Wildlife Is Overlooked in the Epidemiology of Medically Important Antibiotic‐Resistant Bacteria.” Antimicrobial Agents and Chemotherapy 63, no. 8: e01167‐19. 10.1128/AAC.01167-19. 31209001 PMC6658798

[emi470404-bib-0017] Doyle, C. , K. Wall , S. Fanning , and B. J. McMahon . 2025. “Making Sense of Sentinels: Wildlife as the One Health Bridge for Environmental Antimicrobial Resistance Surveillance.” Journal of Applied Microbiology 136, no. 1: lxaf017. 10.1093/jambio/lxaf017. 39805713

[emi470404-bib-0018] Edman‐Wallér, J. , J. Andersson , M. Nelson , et al. 2025. “A Hospital‐Wide Outbreak of Extended‐Spectrum β‐Lactamase‐Producing *Klebsiella oxytoca* Associated With Contaminated Sinks and Associated Plumbing: Outbreak Report, Risk Factor Analysis and Plasmid Mapping.” Journal of Hospital Infection 162: 1–8. 10.1016/j.jhin.2025.05.002. 40393526

[emi470404-bib-0019] EFSA & ECDC . 2024. “The European Union Summary Report on Antimicrobial Resistance in Zoonotic and Indicator Bacteria From Humans, Animals and Food in 2021–2022.” EFSA Journal 22, no. 2: e8583. 10.2903/j.efsa.2024.8583. 38419967 PMC10900121

[emi470404-bib-0020] Egervärn, M. , S. Englund , M. Ljunge , et al. 2017. “Unexpected Common Occurrence of Transferable Extended Spectrum Cephalosporinase‐Producing *Escherichia coli* in Swedish Surface Waters Used for Drinking Water Supply.” Science of the Total Environment 587–588: 466–472. 10.1016/j.scitotenv.2017.02.157. 28258755

[emi470404-bib-0021] Elmarghani, E. D. , R. Spörndly , E. Mourkas , and J. D. Järhult . 2025. “Temporal and Spatial Variation of Third‐Generation Cephalosporin‐Resistant and ESBL‐Producing *Escherichia coli* in a Swedish Urban Water System.” Next Research 2, no. 3: 100436. 10.1016/j.nexres.2025.100436.

[emi470404-bib-0022] Espinosa, L. , A. Gray , G. Duffy , S. Fanning , and B. J. McMahon . 2018. “A Scoping Review on the Prevalence of Shiga‐Toxigenic *Escherichia coli* in Wild Animal Species.” Zoonoses and Public Health 65, no. 8: 911–920. 10.1111/zph.12508. 30099841

[emi470404-bib-0023] FAO & WHO . 2018. Shiga Toxin‐Producing *Escherichia coli* (STEC) and Food: Attribution, Characterization, and Monitoring. Food and Agriculture Organization of the United Nations.

[emi470404-bib-0024] Feldgarden, M. , V. Brover , D. H. Haft , et al. 2019. “Validating the AMRFinder Tool and Resistance Gene Database by Using Antimicrobial Resistance Genotype‐Phenotype Correlations in a Collection of Isolates.” Antimicrobial Agents and Chemotherapy 63, no. 11: 10‐1128. 10.1128/aac.00483-19. PMC6811410 31427293

[emi470404-bib-0025] Fletcher, J. W. J. , S. Tollington , R. Cox , et al. 2025. “Utilisation of Anthropogenic Food by Red Foxes ( *Vulpes vulpes* ) in Britain as Determined by Stable Isotope Analysis.” Ecology and Evolution 15, no. 3: e70844. 10.1002/ece3.70844. 40027416 PMC11868989

[emi470404-bib-0026] Folkhälsomyndigheten & SVA . 2023. “Swedres–Svarm 2023 Sales of Antibiotics and Occurrence.” https://www.sva.se/media/cegdew24/swedres‐svarm‐2023.pdf.

[emi470404-bib-0027] Folkhälsomyndigheten & SVA . 2024. “Swedres–Svarm 2024 Sales of Antibiotics and Occurrence.” https://www.sva.se/media/amupibfr/swedres‐svarm‐2024‐webb.pdf.

[emi470404-bib-0028] Furlan, J. P. R. , M. S. Ramos , R. d. S. Rosa , E. A. Savazzi , and E. G. Stehling . 2022. “Occurrence and Genetic Characteristics of Multidrug‐Resistant *Escherichia coli* Isolates Co‐Harboring Antimicrobial Resistance Genes and Metal Tolerance Genes in Aquatic Ecosystems.” International Journal of Hygiene and Environmental Health 244: 114003. 10.1016/j.ijheh.2022.114003. 35779436

[emi470404-bib-0029] Gordon, D. M. , and A. Cowling . 2003. “The Distribution and Genetic Structure of *Escherichia coli* in Australian Vertebrates: Host and Geographic Effects.” Microbiology 149, no. 12: 3575–3586. 10.1099/mic.0.26486-0. 14663089

[emi470404-bib-0030] Greig, J. , A. Rajić , I. Young , M. Mascarenhas , L. Waddell , and J. LeJeune . 2015. “A Scoping Review of the Role of Wildlife in the Transmission of Bacterial Pathogens and Antimicrobial Resistance to the Food Chain.” Zoonoses and Public Health 62, no. 4: 269–284. 10.1111/zph.12147. 25175882

[emi470404-bib-0031] Guenther, S. , K. Aschenbrenner , I. Stamm , et al. 2012. “Comparable High Rates of Extended‐Spectrum‐Beta‐Lactamase‐Producing *Escherichia coli* in Birds of Prey From Germany and Mongolia.” PLoS One 7, no. 12: e53039. 10.1371/journal.pone.0053039. 23300857 PMC3534101

[emi470404-bib-0032] Guenther, S. , C. Ewers , and L. H. Wieler . 2011. “Extended‐Spectrum Beta‐Lactamases Producing *E. coli* in Wildlife, Yet Another Form of Environmental Pollution?” Frontiers in Microbiology 2: 246. 10.3389/fmicb.2011.00246. 22203818 PMC3244693

[emi470404-bib-0033] Guo, S. , D. Wakeham , H. J. M. Brouwers , et al. 2015. “Human‐Associated Fluoroquinolone‐Resistant *Escherichia coli* Clonal Lineages, Including ST354, Isolated From Canine Feces and Extraintestinal Infections in Australia.” Microbes and Infection 17, no. 4: 266–274. 10.1016/j.micinf.2014.12.016. 25576024

[emi470404-bib-0034] Halaji, M. , A. Fayyazi , M. Rajabnia , D. Zare , A. Pournajaf , and R. Ranjbar . 2022. “Phylogenetic Group Distribution of Uropathogenic Escherichia Coli and Related Antimicrobial Resistance Pattern: A Meta‐Analysis and Systematic Review.” Frontiers in Cellular and Infection Microbiology 12: 790184. 10.3389/fcimb.2022.790184. 35281449 PMC8914322

[emi470404-bib-0035] Hessman, J. , C. Atterby , B. Olsen , and J. D. Järhult . 2018. “High Prevalence and Temporal Variation of Extended Spectrum β‐Lactamase–Producing Bacteria in Urban Swedish Mallards.” Microbial Drug Resistance 24, no. 6: 822–829. 10.1089/mdr.2017.0263. 29304312

[emi470404-bib-0036] Höcketstaller, K. , I. A. Marti , C. Bank , B. Yilmaz , and J. Becker . 2025. “Antimicrobial Resistance in Wildlife: Associations With Environmental Factors and Taxonomic Variation.” Environmental Research 281: 121968. 10.1016/j.envres.2025.121968. 40436191

[emi470404-bib-0037] Homeier‐Bachmann, T. , A. K. Schütz , S. Dreyer , J. Glanz , K. Schaufler , and F. J. Conraths . 2022. “Genomic Analysis of ESBL‐Producing *E. coli* in Wildlife From North‐Eastern Germany.” Antibiotics 11, no. 2: 123. 10.3390/antibiotics11020123. 35203726 PMC8868512

[emi470404-bib-0038] Jobbins, S. E. , and K. A. Alexander . 2015. “From Whence They Came—Antibiotic‐Resistant *Escherichia coli* in African Wildlife.” Journal of Wildlife Diseases 51, no. 4: 811–820. 10.7589/2014-11-257. 26221860

[emi470404-bib-0039] Joensen, K. G. , F. Scheutz , O. Lund , et al. 2014. “Real‐Time Whole‐Genome Sequencing for Routine Typing, Surveillance, and Outbreak Detection of Verotoxigenic *Escherichia coli* .” Journal of Clinical Microbiology 52, no. 5: 1501–1510. 10.1128/jcm.03617-13. 24574290 PMC3993690

[emi470404-bib-0040] Johnson, J. R. , B. D. Johnston , S. B. Porter , et al. 2019. “Rapid Emergence, Subsidence, and Molecular Detection of *Escherichia coli* Sequence Type 1193‐fimH64, a New Disseminated Multidrug‐Resistant Commensal and Extraintestinal Pathogen.” Journal of Clinical Microbiology 57: 10‐1128. 10.1128/jcm.01664-18. PMC6498021 30787145

[emi470404-bib-0041] Johnson, J. R. , V. Tchesnokova , B. Johnston , et al. 2013. “Abrupt Emergence of a Single Dominant Multidrug‐Resistant Strain of *Escherichia coli* .” Journal of Infectious Diseases 207, no. 6: 919–928. 10.1093/infdis/jis933. 23288927 PMC3571447

[emi470404-bib-0042] Köhler, C.‐D. , and U. Dobrindt . 2011. “What Defines Extraintestinal Pathogenic *Escherichia coli* ?” International Journal of Medical Microbiology 301, no. 8: 642–647. 10.1016/j.ijmm.2011.09.006. 21982038

[emi470404-bib-0043] Kozoderović, G. , D. Todorović , M. Đilas , B. Kartalović , and M. Velhner . 2021. “White‐Tailed Eagles ( *Haliaeetus albicilla* ) in Protected Danube Wetlands as Carriers of *Escherichia coli* With Resistance and Virulence Genes.” European Journal of Wildlife Research 67, no. 6: 103. 10.1007/s10344-021-01547-6.

[emi470404-bib-0044] Laborda, P. , F. Sanz‐García , L. E. Ochoa‐Sánchez , T. Gil‐Gil , S. Hernando‐Amado , and J. L. Martínez . 2022. “Wildlife and Antibiotic Resistance.” Frontiers in Cellular and Infection Microbiology 12: 873989. 10.3389/fcimb.2022.873989. 35646736 PMC9130706

[emi470404-bib-0045] Lagerstrom, K. M. , and E. A. Hadly . 2021. “The Under‐Investigated Wild Side of *Escherichia coli* : Genetic Diversity, Pathogenicity and Antimicrobial Resistance in Wild Animals.” Proceedings of the Royal Society B: Biological Sciences 288, no. 1948: 20210399. 10.1098/rspb.2021.0399. PMC8059539 33849316

[emi470404-bib-0046] Lagerstrom, K. M. , and E. A. Hadly . 2023. “Under‐Appreciated Phylogroup Diversity of *Escherichia coli* Within and Between Animals at the Urban‐Wildland Interface.” Applied and Environmental Microbiology 89, no. 6: e00142‐23. 10.1128/aem.00142-23. 37191541 PMC10305377

[emi470404-bib-0047] Larsen, J. , C. L. Raisen , X. Ba , et al. 2022. “Emergence of Methicillin Resistance Predates the Clinical Use of Antibiotics.” Nature 602, no. 7895: 135–141. 10.1038/s41586-021-04265-w. 34987223 PMC8810379

[emi470404-bib-0048] Lau, S. H. , S. Reddy , J. Cheesbrough , et al. 2008. “Major Uropathogenic *Escherichia coli* Strain Isolated in the Northwest of England Identified by Multilocus Sequence Typing.” Journal of Clinical Microbiology 46, no. 3: 1076–1080. 10.1128/jcm.02065-07. 18199778 PMC2268376

[emi470404-bib-0049] Le Gall, T. , O. Clermont , S. Gouriou , et al. 2007. “Extraintestinal Virulence Is a Coincidental by‐Product of Commensalism in B2 Phylogenetic Group *Escherichia coli* Strains.” Molecular Biology and Evolution 24, no. 11: 2373–2384. 10.1093/molbev/msm172. 17709333

[emi470404-bib-0050] Lee, S. , P. Fan , T. Liu , et al. 2022. “Transmission of Antibiotic Resistance at the Wildlife‐Livestock Interface.” Communications Biology 5, no. 1: 585. 10.1038/s42003-022-03520-8. 35705693 PMC9200806

[emi470404-bib-0051] Literak, I. , M. Dolejska , T. Radimersky , et al. 2010. “Antimicrobial‐Resistant Faecal *Escherichia coli* in Wild Mammals in Central Europe: Multiresistant *Escherichia coli* Producing Extended‐Spectrum Beta‐Lactamases in Wild Boars.” Journal of Applied Microbiology 108, no. 5: 1702–1711. 10.1111/j.1365-2672.2009.04572.x. 19849769

[emi470404-bib-0052] Liu, S. , D. Jin , R. Lan , et al. 2015. “Escherichia Marmotae sp. Nov., Isolated From Faeces of *Marmota himalayana* .” International Journal of Systematic and Evolutionary Microbiology 65, no. Pt_7: 2130–2134. 10.1099/ijs.0.000228. 25851592

[emi470404-bib-0053] Llorente, M. T. , R. Escudero , R. Ramiro , et al. 2023. “Enteroaggregative *Escherichia coli* as Etiological Agent of Endemic Diarrhea in Spain: A Prospective Multicenter Prevalence Study With Molecular Characterization of Isolates.” Frontiers in Microbiology 14: 1120285. 10.3389/fmicb.2023.1120285. 37065134 PMC10100739

[emi470404-bib-0054] Loy, A. , A. Kranz , A. Oleynikov , A. Roos , M. Savage , and N. Duplaix . 2022. “ *Lutra lutra* (Amended Version of 2021 Assessment).” In The IUCN Red List of Threatened Species. International Union for Conservation of Nature (IUCN). 10.2305/IUCN.UK.2022-2.RLTS.T12419A218069689.en.

[emi470404-bib-0055] Luo, Q. , W. Yu , K. Zhou , et al. 2017. “Molecular Epidemiology and Colistin Resistant Mechanism of Mcr‐Positive and Mcr‐Negative Clinical Isolated *Escherichia coli* .” Frontiers in Microbiology 8: 2262. 10.3389/fmicb.2017.02262. 29250039 PMC5715374

[emi470404-bib-0056] Massot, M. , A.‐S. Daubié , O. Clermont , et al. 2016. “Phylogenetic, Virulence and Antibiotic Resistance Characteristics of Commensal Strain Populations of *Escherichia coli* From Community Subjects in the Paris Area in 2010 and Evolution Over 30 Years.” Microbiology 162, no. 4: 642–650. 10.1099/mic.0.000242. 26822436 PMC6365622

[emi470404-bib-0057] Masters, N. , M. Christie , H. Stratton , and M. Katouli . 2015. “Viability and Stability of *Escherichia coli* and Enterococci Populations in Fecal Samples Upon Freezing.” Canadian Journal of Microbiology 61, no. 7: 495–501. 10.1139/cjm-2015-0020. 26053765

[emi470404-bib-0058] Ménard, L.‐P. , and J. D. Dubreuil . 2002. “Enteroaggregative *Escherichia coli* Heat‐Stable Enterotoxin 1 (EAST1): A New Toxin With an Old Twist.” Critical Reviews in Microbiology 28, no. 1: 43–60. 10.1080/1040-840291046687. 12003040

[emi470404-bib-0059] Mengistu, T. S. , B. Garcias , G. Castellanos , C. Seminati , R. A. Molina‐López , and L. Darwich . 2022. “Occurrence of Multidrug Resistant Gram‐Negative Bacteria and Resistance Genes in Semi‐Aquatic Wildlife— *Trachemys scripta* , *Neovison vison* and *Lutra lutra* —As Sentinels of Environmental Health.” Science of the Total Environment 830: 154814. 10.1016/j.scitotenv.2022.154814. 35341839

[emi470404-bib-0060] Micenková, L. , J. Bosák , M. Vrba , A. Ševčíková , and D. Šmajs . 2016. “Human Extraintestinal Pathogenic *Escherichia coli* Strains Differ in Prevalence of Virulence Factors, Phylogroups, and Bacteriocin Determinants.” BMC Microbiology 16, no. 1: 218. 10.1186/s12866-016-0835-z. 27646192 PMC5028950

[emi470404-bib-0061] Mo, S. S. , A. M. Urdahl , K. Madslien , et al. 2018. “What Does the Fox Say? Monitoring Antimicrobial Resistance in the Environment Using Wild Red Foxes as an Indicator.” PLoS One 13, no. 5: e0198019. 10.1371/journal.pone.0198019. 29799852 PMC5969755

[emi470404-bib-0062] Modgil, V. , H. Kaur , J. Mahindroo , B. Mohan , and N. Taneja . 2024. “Sequence Types of Enteroaggregative *Escherichia coli* Strains Recovered From Human, Animal, and Environmental Sources: India.” Gene Reports 37: 102017. 10.1016/j.genrep.2024.102017.

[emi470404-bib-0063] Mughini‐Gras, L. , A. Dorado‐García , E. van Duijkeren , et al. 2019. “Attributable Sources of Community‐Acquired Carriage of *Escherichia coli* Containing β‐Lactam Antibiotic Resistance Genes: A Population‐Based Modelling Study.” Lancet Planetary health 3, no. 8: e357–e369. 10.1016/S2542-5196(19)30130-5. 31439317

[emi470404-bib-0064] Nesporova, K. , M. Ruzickova , H. Tarabai , et al. 2024. “Changing Dynamics of Antibiotic Resistant *Escherichia* in Caspian Gulls Shows the Importance of Longitudinal Environmental Studies.” Environment International 186: 108606. 10.1016/j.envint.2024.108606. 38554502

[emi470404-bib-0065] Ny, S. , S. Löfmark , S. Börjesson , et al. 2017. “Community Carriage of ESBL‐Producing *Escherichia coli* Is Associated With Strains of Low Pathogenicity: A Swedish Nationwide Study.” Journal of Antimicrobial Chemotherapy 72, no. 2: 582–588. 10.1093/jac/dkw419. 27798205

[emi470404-bib-0066] O'Hagan, M. J. H. , A. V. Pascual‐Linaza , C. Couzens , et al. 2021. “Estimation of the Prevalence of Antimicrobial Resistance in Badgers ( *Meles meles* ) and Foxes ( *Vulpes vulpes* ) in Northern Ireland.” Frontiers in Microbiology 12: 596891. 10.3389/fmicb.2021.596891. 33679630 PMC7930819

[emi470404-bib-0067] Osińska, M. , A. Nowakiewicz , P. Zięba , S. Gnat , D. Łagowski , and A. Trościańczyk . 2022. “A Rich Mosaic of Resistance in Extended‐Spectrum β‐Lactamase‐Producing *Escherichia coli* Isolated From Red Foxes ( *Vulpes vulpes* ) in Poland as a Potential Effect of Increasing Synanthropization.” Science of the Total Environment 818: 151834. 10.1016/j.scitotenv.2021.151834. 34808162

[emi470404-bib-0068] Petitjean, M. , B. Condamine , C. Burdet , E. Denamur , and E. Ruppé . 2021. “Phylum Barrier and *Escherichia coli* Intra‐Species Phylogeny Drive the Acquisition of Antibiotic‐Resistance Genes.” Microbial Genomics 7, no. 8: 000489. 10.1099/mgen.0.000489. 34435947 PMC8549366

[emi470404-bib-0069] Pinto, L. , H. Radhouani , C. Coelho , et al. 2010. “Genetic Detection of Extended‐Spectrum β‐Lactamase‐Containing *Escherichia coli* Isolates From Birds of Prey From Serra da Estrela Natural Reserve in Portugal.” Applied and Environmental Microbiology 76, no. 12: 4118–4120. 10.1128/AEM.02761-09. 20418435 PMC2893492

[emi470404-bib-0070] Pitout, J. D. D. , G. Peirano , L. Chen , R. DeVinney , and Y. Matsumura . 2022. “ *Escherichia coli* ST1193: Following in the Footsteps of *E. coli* ST131.” Antimicrobial Agents and Chemotherapy 66, no. 7: e00511‐22. 10.1128/aac.00511-22. 35658504 PMC9295538

[emi470404-bib-0071] Price, L. B. , J. R. Johnson , M. Aziz , et al. 2013. “The Epidemic of Extended‐Spectrum‐β‐Lactamase‐Producing *Escherichia coli* ST131 Is Driven by a Single Highly Pathogenic Subclone, *H* 30‐Rx.” mBio 4, no. 6: e00377‐13. 10.1128/mBio.00377-13. 24345742 PMC3870262

[emi470404-bib-0072] Radhouani, H. , P. Poeta , A. Gonçalves , R. Pacheco , R. Sargo , and G. Igrejas . 2012. “Wild Birds as Biological Indicators of Environmental Pollution: Antimicrobial Resistance Patterns of Escherichia Coli and Enterococci Isolated From Common Buzzards ( *Buteo buteo* ).” Journal of Medical Microbiology 61, no. 6: 837–843. 10.1099/jmm.0.038364-0. 22403140

[emi470404-bib-0073] Radhouani, H. , N. Silva , P. Poeta , C. Torres , S. Correia , and G. Igrejas . 2014. “Potential Impact of Antimicrobial Resistance in Wildlife, Environment and Human Health.” Frontiers in Microbiology 5: 23. 10.3389/fmicb.2014.00023. 24550896 PMC3913889

[emi470404-bib-0074] Ramey, A. M. , and C. A. Ahlstrom . 2020. “Antibiotic Resistant Bacteria in Wildlife: Perspectives on Trends, Acquision and Dissemination, Data Gaps and Future Directions.” Journal of Wildlife Diseases 56, no. 1: 1. 10.7589/2019-04-099. 31567035

[emi470404-bib-0075] Richter, M. , R. Rosselló‐Móra , F. Oliver Glöckner , and J. Peplies . 2016. “JSpeciesWS: A Web Server for Prokaryotic Species Circumscription Based on Pairwise Genome Comparison.” Bioinformatics 32, no. 6: 929–931. 10.1093/bioinformatics/btv681. 26576653 PMC5939971

[emi470404-bib-0076] Saied, A. A. 2024. “Integrating Wildlife Within National Action Plan for Antimicrobial Resistance.” New Microbes New Infections 62: 101467. 10.1016/j.nmni.2024.101467. 39282144 PMC11400982

[emi470404-bib-0077] Sharma, P. , S. Maherchandani , B. N. Shringi , S. K. Kashyap , and K. S. G. Sundar . 2018. “Temporal Variations in Patterns of *Escherichia coli* Strain Diversity and Antimicrobial Resistance in the Migrant Egyptian Vulture.” Infection Ecology & Epidemiology 8, no. 1: 1450590. 10.1080/20008686.2018.1450590. 29755700 PMC5941391

[emi470404-bib-0078] Simões, R. , C. Ferreira , J. Gonçalves , et al. 2012. “Occurrence of Virulence Genes in Multidrug‐Resistant *Escherichia coli* Isolates From Iberian Wolves ( *Canis lupus* Signatus) in Portugal.” European Journal of Wildlife Research 58, no. 4: 677–684. 10.1007/s10344-012-0616-4.

[emi470404-bib-0079] Sivertsen, A. , R. Dyrhovden , M. G. Tellevik , et al. 2022. “Escherichia Marmotae—A Human Pathogen Easily Misidentified as *Escherichia coli* .” Microbiology Spectrum 10, no. 2: e02035‐21. 10.1128/spectrum.02035-21. PMC9045135 35380461

[emi470404-bib-0080] Söderlund, R. , H. Skarin , S. Börjesson , et al. 2019. “Prevalence and Genomic Characteristics of Zoonotic Gastro‐Intestinal Pathogens and ESBL/pAmpC Producing *Enterobacteriaceae* Among Swedish Corvid Birds.” Infection Ecology & Epidemiology 9, no. 1: 1701399. 10.1080/20008686.2019.1701399. 32002147 PMC6968639

[emi470404-bib-0081] Ståhlberg, S. , E. Bassi , V. Viviani , and M. Apollonio . 2017. “Quantifying Prey Selection of Northern and Southern European Wolves ( *Canis lupus* ).” Mammalian Biology 83: 34–43. 10.1016/j.mambio.2016.11.001.

[emi470404-bib-0082] SVA . 2022. “Årsredovisning 2022.” https://www.sva.se/media/obdpdptx/svakom00032022‐sva‐aarsrapport‐2022.pdf.

[emi470404-bib-0083] SVA . 2023. Wildlife Disease Surveillance in Sweden 2022.

[emi470404-bib-0084] Tenaillon, O. , D. Skurnik , B. Picard , and E. Denamur . 2010. “The Population Genetics of Commensal *Escherichia coli* .” Nature Reviews Microbiology 8, no. 3: 207–217. 10.1038/nrmicro2298. 20157339

[emi470404-bib-0085] Torres, R. T. , and C. Fonseca . 2016. “Perspectives on the Iberian Wolf in Portugal: Population Trends and Conservation Threats.” Biodiversity and Conservation 25, no. 3: 411–425. 10.1007/s10531-016-1061-6.

[emi470404-bib-0086] Van Nederveen, V. , and A. R. Melton‐Celsa . 2025. “Enteroaggregative *Escherichia coli* (EAEC).” EcoSal Plus 13, no. 1: eesp‐0011‐2024. 10.1128/ecosalplus.esp-0011-2024. PMC12707135 40586580

[emi470404-bib-0087] Videvall, E. , M. Strandh , A. Engelbrecht , S. Cloete , and C. K. Cornwallis . 2018. “Measuring the Gut Microbiome in Birds: Comparison of Faecal and Cloacal Sampling.” Molecular Ecology Resources 18, no. 3: 424–434. 10.1111/1755-0998.12744. 29205893

[emi470404-bib-0088] Wallensten, A. , J. Hernandez , K. Ardiles , D. Gonzalez‐Acuna , M. Drobni , and B. Olsen . 2011. “Extended Spectrum Beta‐Lactamases Detected in *Escherichia coli* From Gulls in Stockholm, Sweden.” Infection Ecology & Epidemiology 1, no. 1: 7030. 10.3402/iee.v1i0.7030. PMC3426345 22957123

[emi470404-bib-0089] Walton, Z. , G. Samelius , M. Odden , and T. Willebrand . 2017. “Variation in Home Range Size of Red Foxes *Vulpes vulpes* Along a Gradient of Productivity and Human Landscape Alteration.” PLoS One 12, no. 4: e0175291. 10.1371/journal.pone.0175291. 28384313 PMC5383297

[emi470404-bib-0090] Wang, M.‐C. , Y.‐H. Fan , Y.‐Z. Zhang , et al. 2023. “Characterization of Uropathogenic *Escherichia coli* Phylogroups Associated With Antimicrobial Resistance, Virulence Factor Distribution, and Virulence‐Related Phenotypes.” Infection, Genetics and Evolution 114: 105493. 10.1016/j.meegid.2023.105493. 37634856

[emi470404-bib-0091] WHO . 2021. WHO Integrated Global Surveillance on ESBL‐Producing *E. coli* Using a One Health Approach: Implementation and Opportunities. 1st ed. World Health Organization.

[emi470404-bib-0092] Woksepp, H. , K. Karlsson , S. Börjesson , O. Karlsson Lindsjö , R. Söderlund , and J. Bonnedahl . 2023. “Dissemination of Carbapenemase‐Producing Enterobacterales Through Wastewater and Gulls at a Wastewater Treatment Plant in Sweden.” Science of the Total Environment 886: 163997. 10.1016/j.scitotenv.2023.163997. 37164093

[emi470404-bib-0093] Wyrsch, E. R. , K. Nesporova , H. Tarabai , et al. 2022. “Urban Wildlife Crisis: Australian Silver Gull Is a Bystander Host to Widespread Clinical Antibiotic Resistance.” mSystems 7, no. 3: e00158‐22. 10.1128/msystems.00158-22. 35469421 PMC9238384

[emi470404-bib-0094] Zhou, Z. , N.‐F. Alikhan , K. Mohamed , et al. 2020. “The EnteroBase User's Guide, With Case Studies on Salmonella Transmissions, *Yersinia pestis* Phylogeny, and Escherichia Core Genomic Diversity.” Genome Research 30, no. 1: 138–152. 10.1101/gr.251678.119. 31809257 PMC6961584

[emi470404-bib-0095] Zhou, Z. , J. Charlesworth , and M. Achtman . 2021. “HierCC: A Multi‐Level Clustering Scheme for Population Assignments Based on Core Genome MLST.” Bioinformatics 37, no. 20: 3645–3646. 10.1093/bioinformatics/btab234. 33823553 PMC8545296

