# Supplementary material for: Using Ongoing Wildlife Surveillance Programs to Monitor Antibiotic Resistant Bacteria in the Environment
Source: Environ Microbiol Rep. 2026 Aug 16;18(4):e70404. doi: 10.1111/1758-2229.70404 (PMC13477815; doi:10.1111/1758-2229.70404)
Supplement: Supplementary file 1 [file EMI4-18-e70404-s002.pdf]

Supplemental Information 2 for:

Using ongoing wildlife surveillance programs to monitor antibiotic resistant bacteria in the environment.

María Rincón-Gracia, Stefan Börjesson, Robert Söderlund, Hanna Woksepp, Aleksija Neimanis, Henrik Uhlhorn, Thomas Rosendal, David Winlund, Hugo Uddströmer, Josef D. Järhult and Jonas Bonnedahl.

Supplemental information 2 – Minimum spanning trees based on cgMLST and HierCC analysis of ESBL- and pAmpC-producing isolates, and of ST88 isolates.

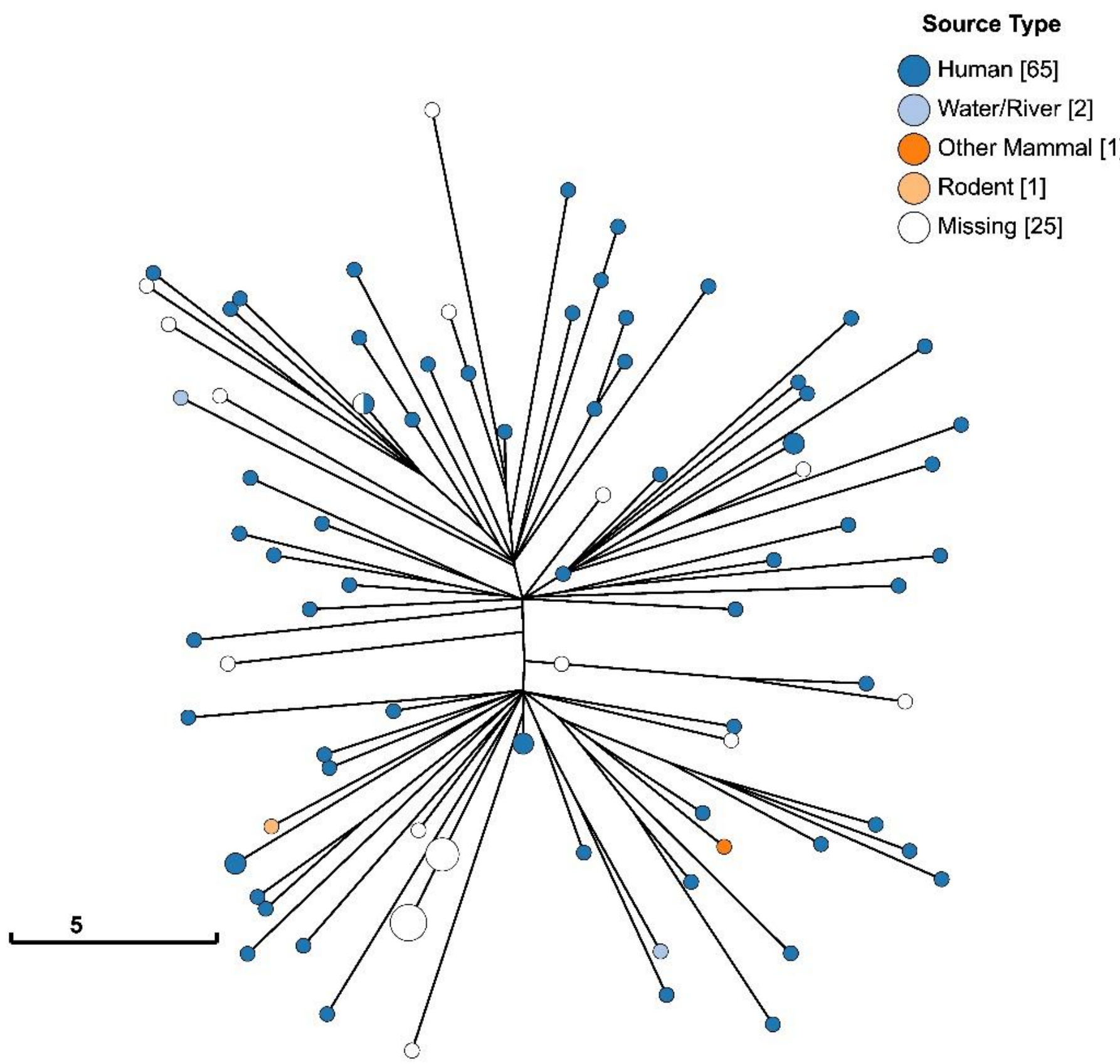

Supplementary Figure S1. Minimum spanning tree based on isolates clustered in HC10 4073. Colored nodes are based on source type. The number of isolates per source type is shown in parentheses [n]. The ESBL-producing isolate (R7b) is represented as Other Mammal.

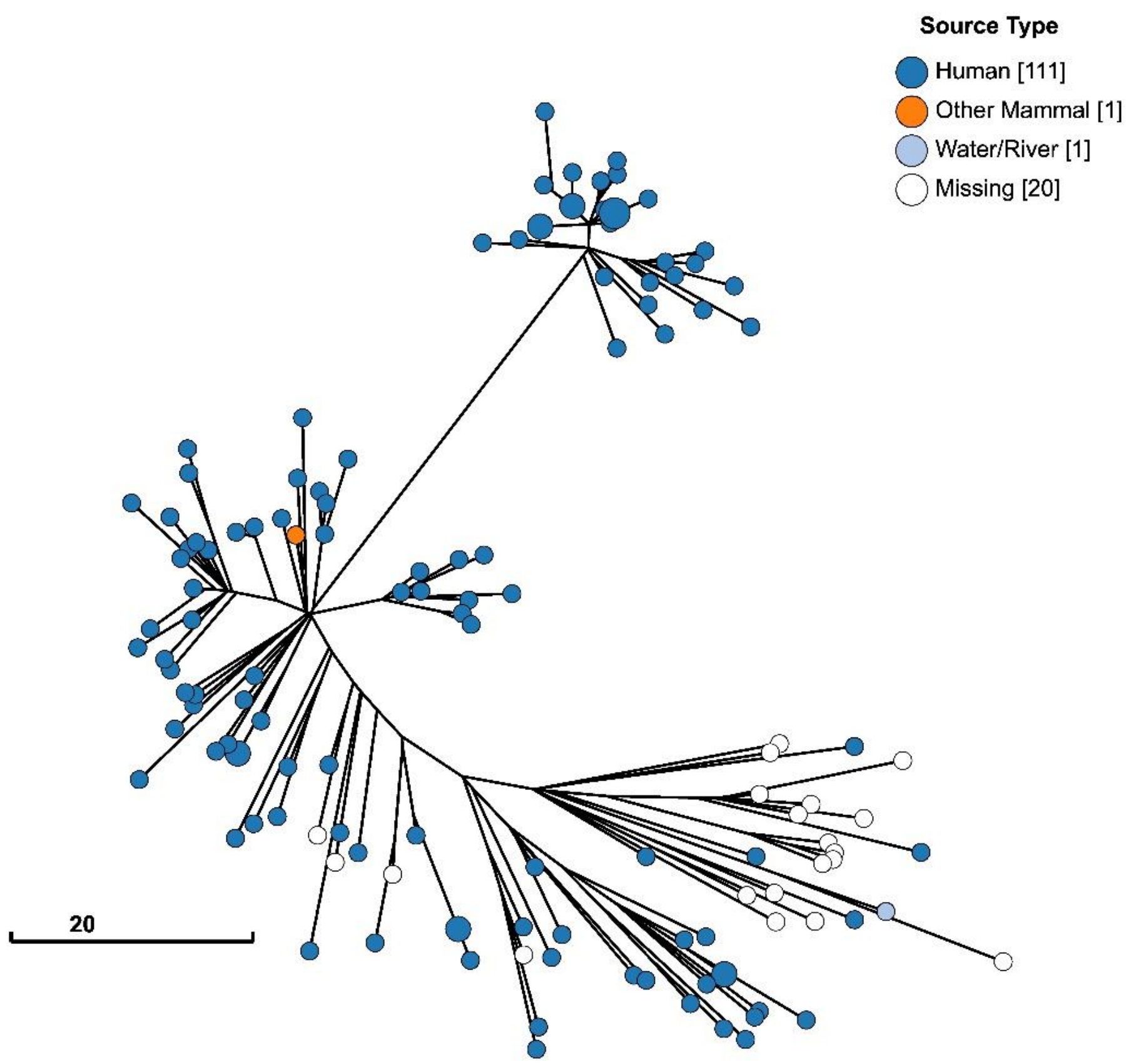

Supplementary Figure S2. Minimum spanning tree based on isolates clustered in HC50 21668. Colored nodes are based on source type. The number of isolates per source type is shown in parentheses [n]. The pAmpC-producing isolate (R8b) is represented as Other Mammal.

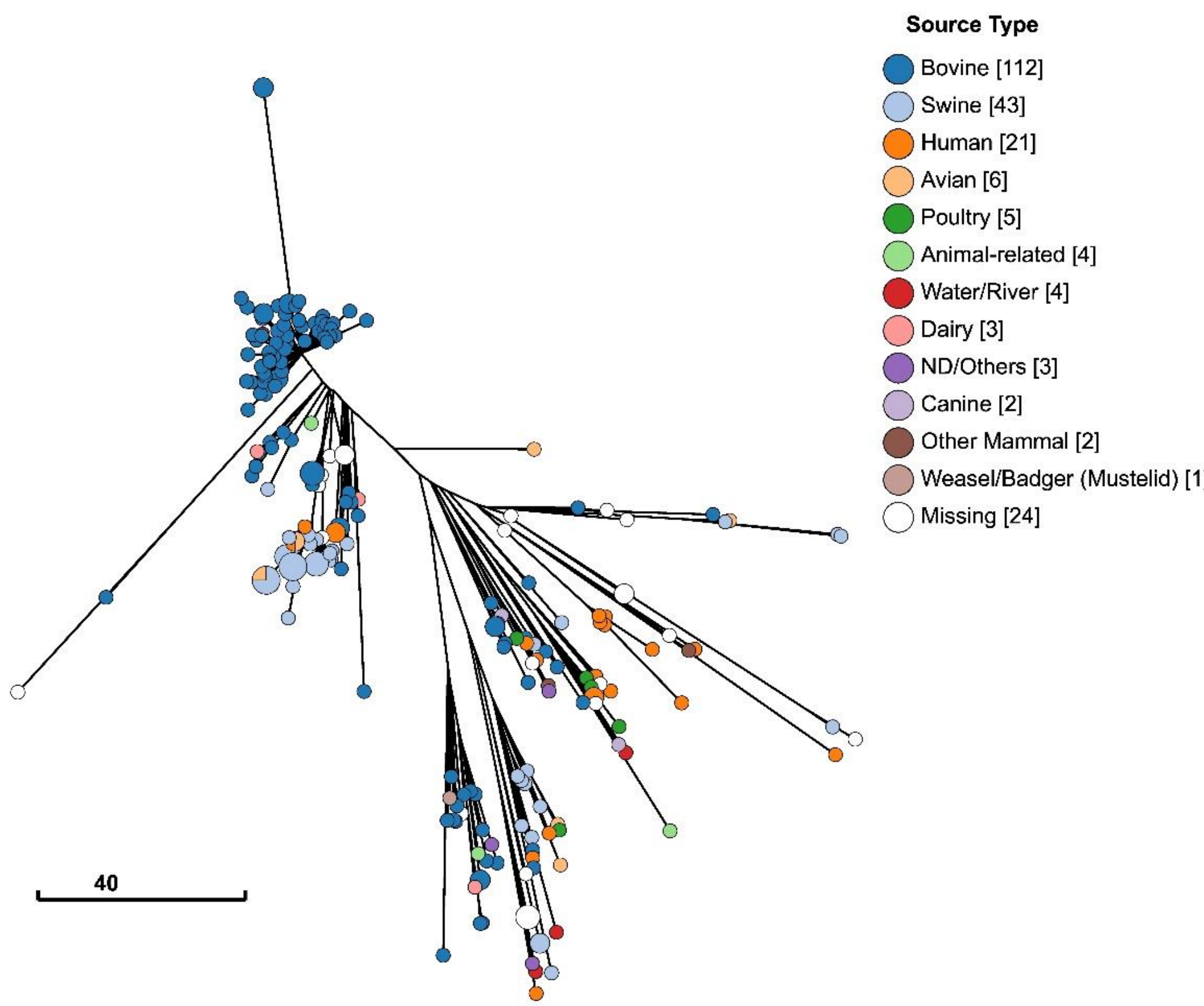

Supplementary Figure S3. Minimum spanning tree based on isolates clustered in HC100 30962. Colored nodes are based on source type. The number of isolates per source type is shown in parentheses [n]. Isolates belonging to ST88 (1031a1Bj and R6a) are represented as Other Mammal.
