# Supplementary material for: Using Ongoing Wildlife Surveillance Programs to Monitor Antibiotic Resistant Bacteria in the Environment
Source: Environ Microbiol Rep. 2026 Aug 16;18(4):e70404. doi: 10.1111/1758-2229.70404 (PMC13477815; doi:10.1111/1758-2229.70404)
Supplement: Supplementary file 2 [file EMI4-18-e70404-s003.pdf]

Supplemental Information 5 for:

Using ongoing wildlife surveillance programs to monitor antibiotic resistant bacteria in the environment.

María Rincón-Gracia, Stefan Börjesson, Robert Söderlund, Hanna Woksepp, Aleksija Neimanis, Henrik Uhlhorn, Thomas Rosendal, David Winlund, Hugo Uddströmer, Josef D. Järhult and Jonas Bonnedahl.

Supplemental information 5 – Sample origin per animal species for samples positive for indicator *E. coli* and correlation with human population density.

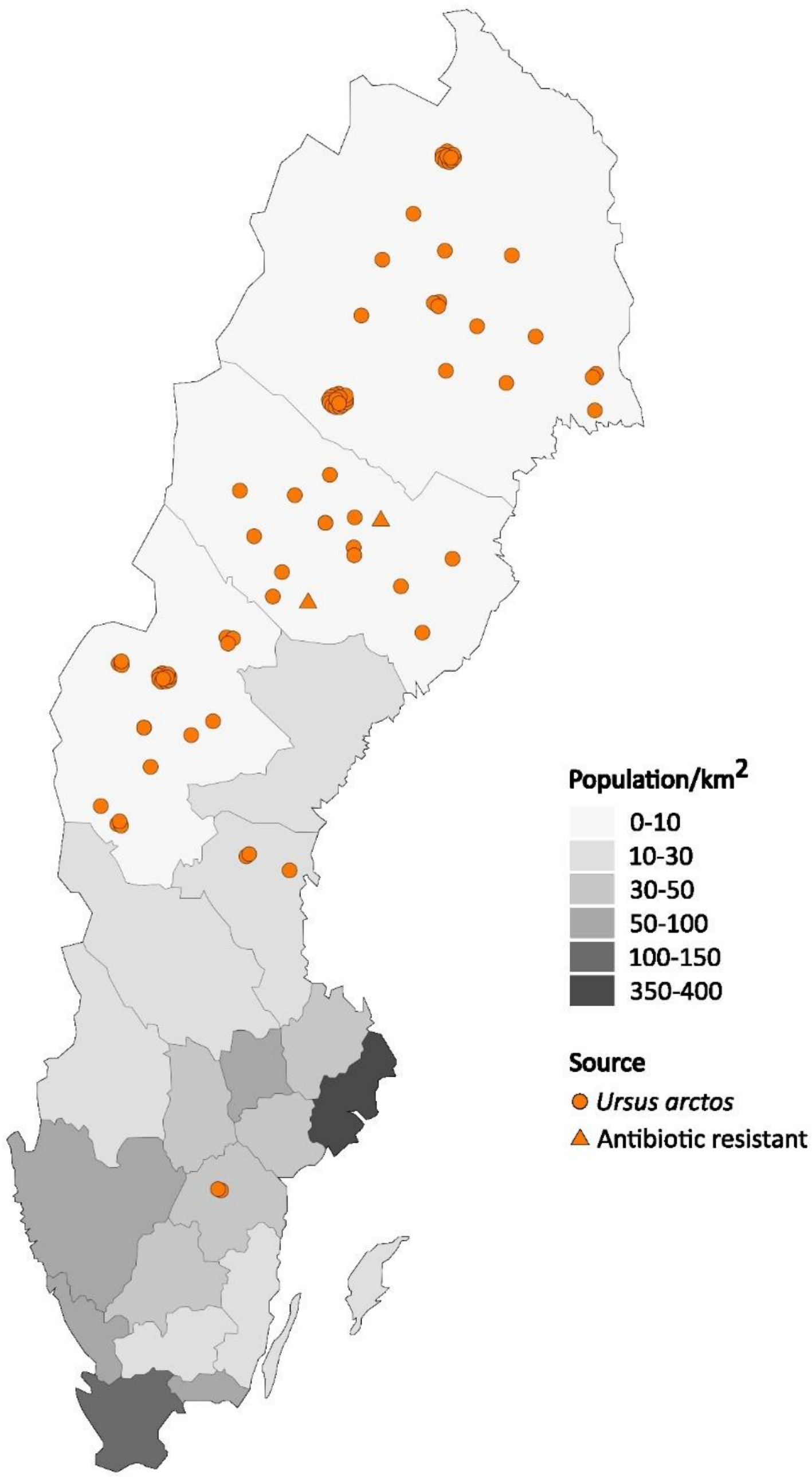

Supplementary Figure S4. Sample origin of bear samples and human population density per county. Information on the geographical origin of two samples was not available (not represented in the figure). Information on municipality was not available for nine of the bear samples (represented in the county they originated from).

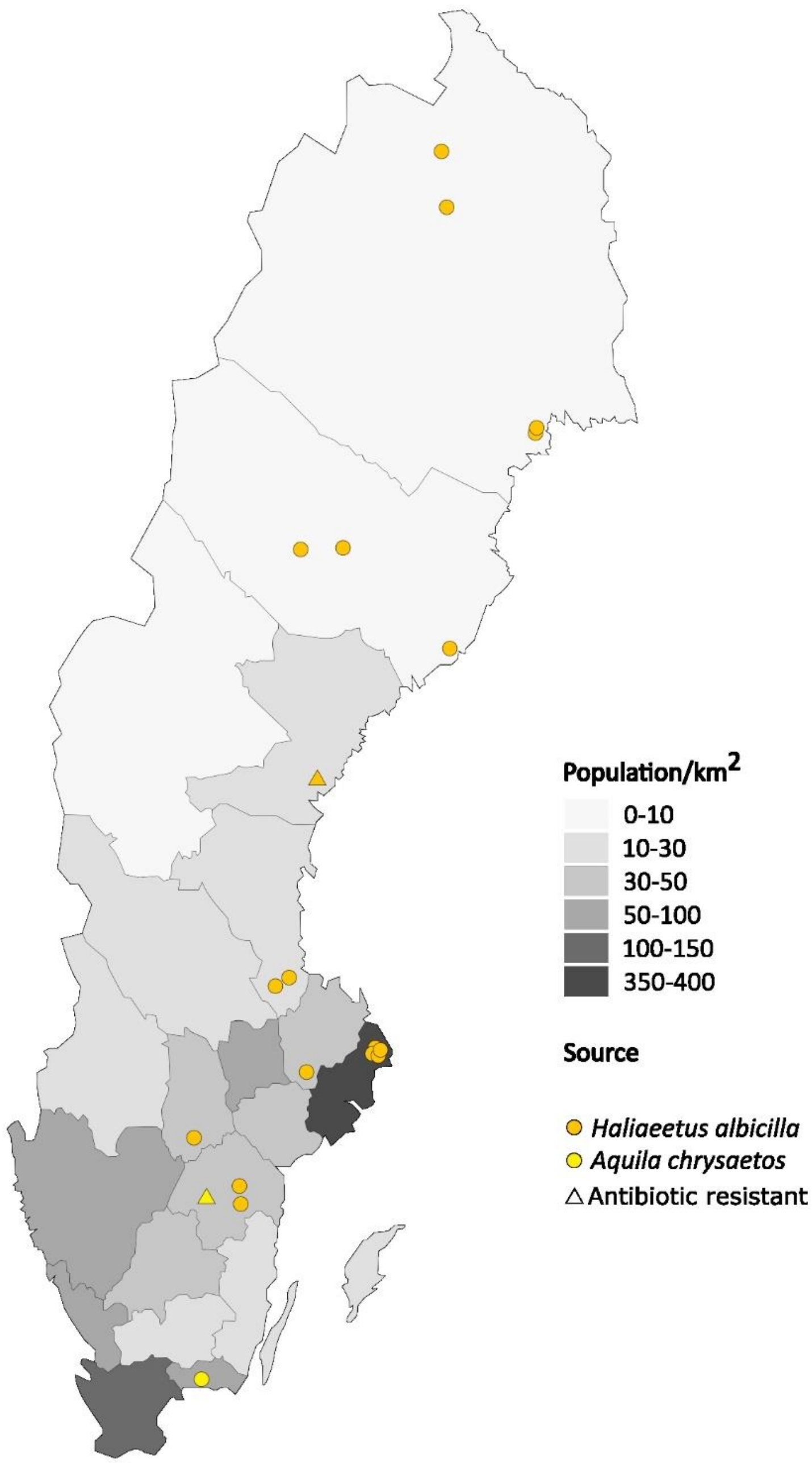

Supplementary Figure S5. Sample origin of eagle samples and population density per county.

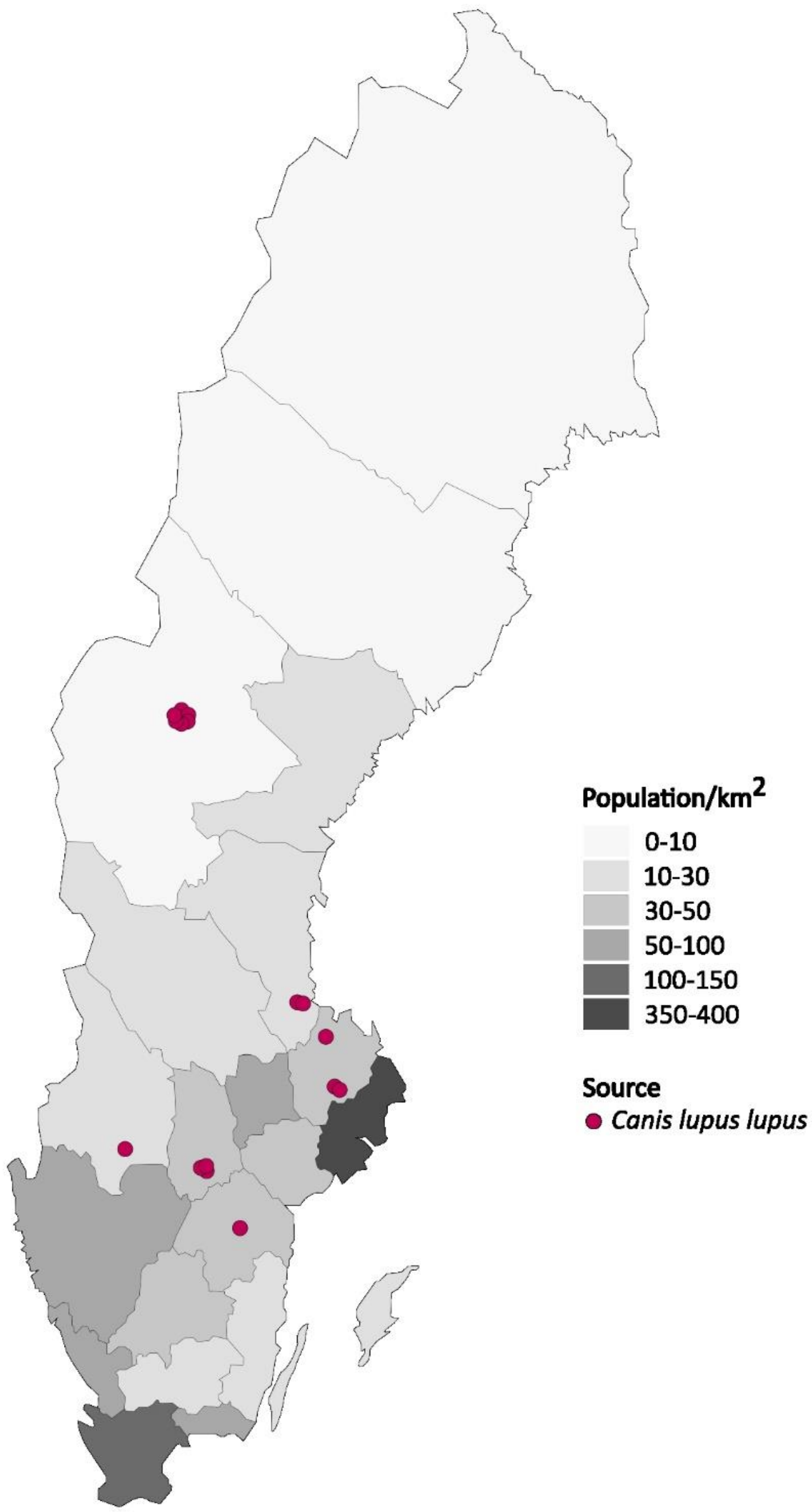

Supplementary Figure S6. Geographical origin of wolf samples and human population density per county. Information on geographical origin was not available for one of the samples (not represented in the figure).

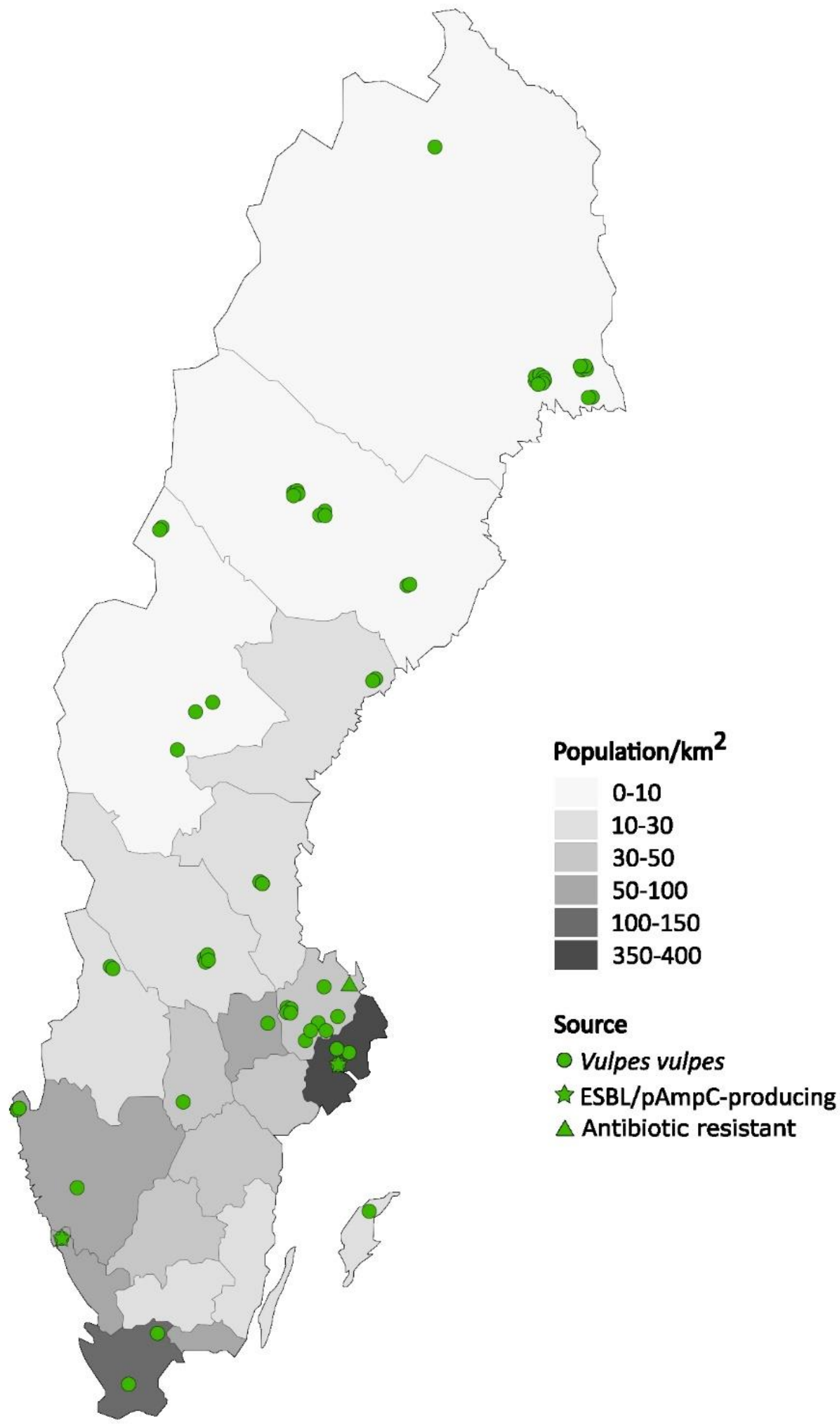

Supplementary Figure S7. Geographical origin of fox samples and human population density per county. ESBL-, pAmpC-producing and antibiotic-resistant isolates are marked according to the legend.

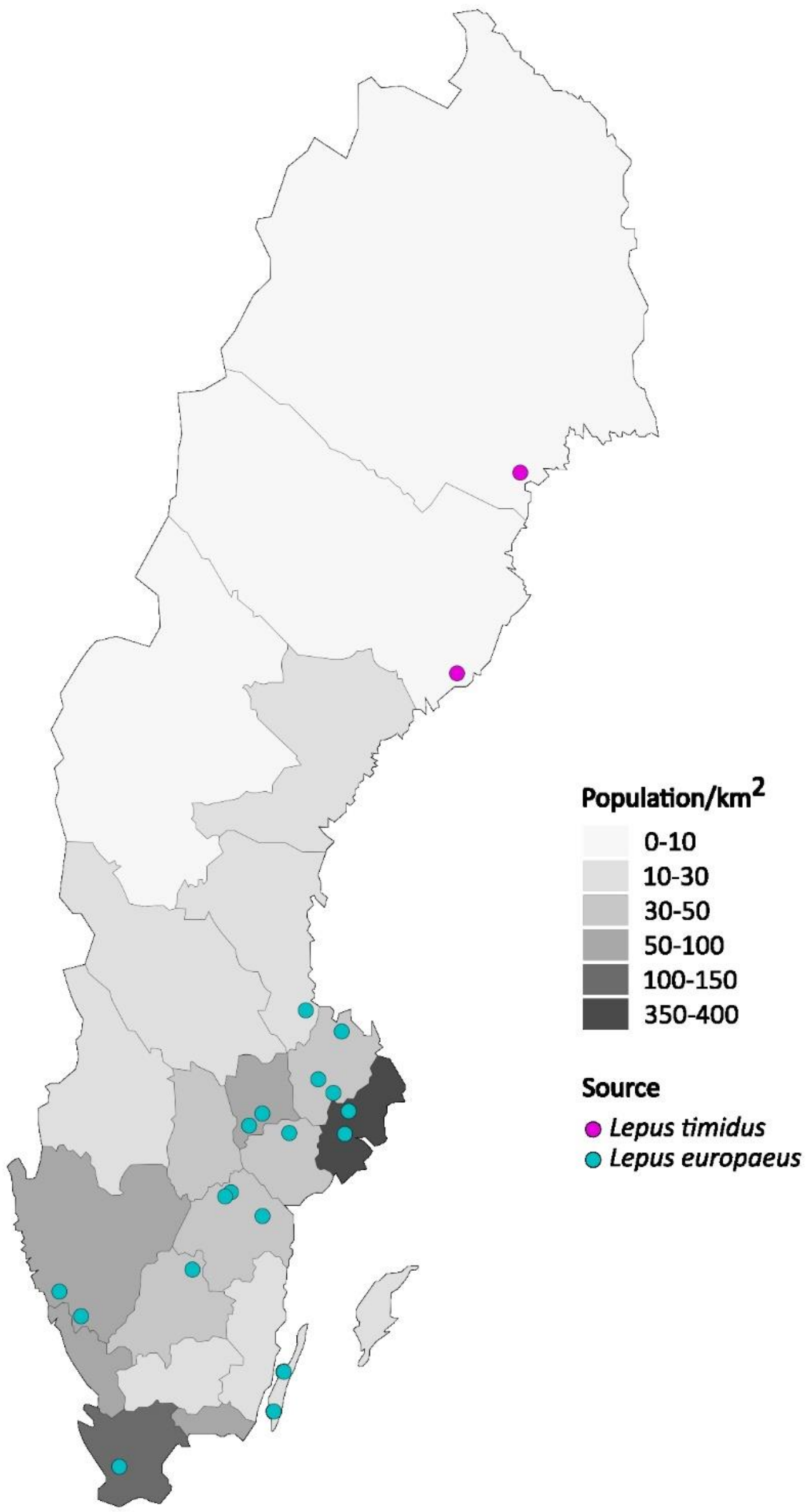

Supplementary Figure S8. Geographical origin of hare samples and human population density per county.

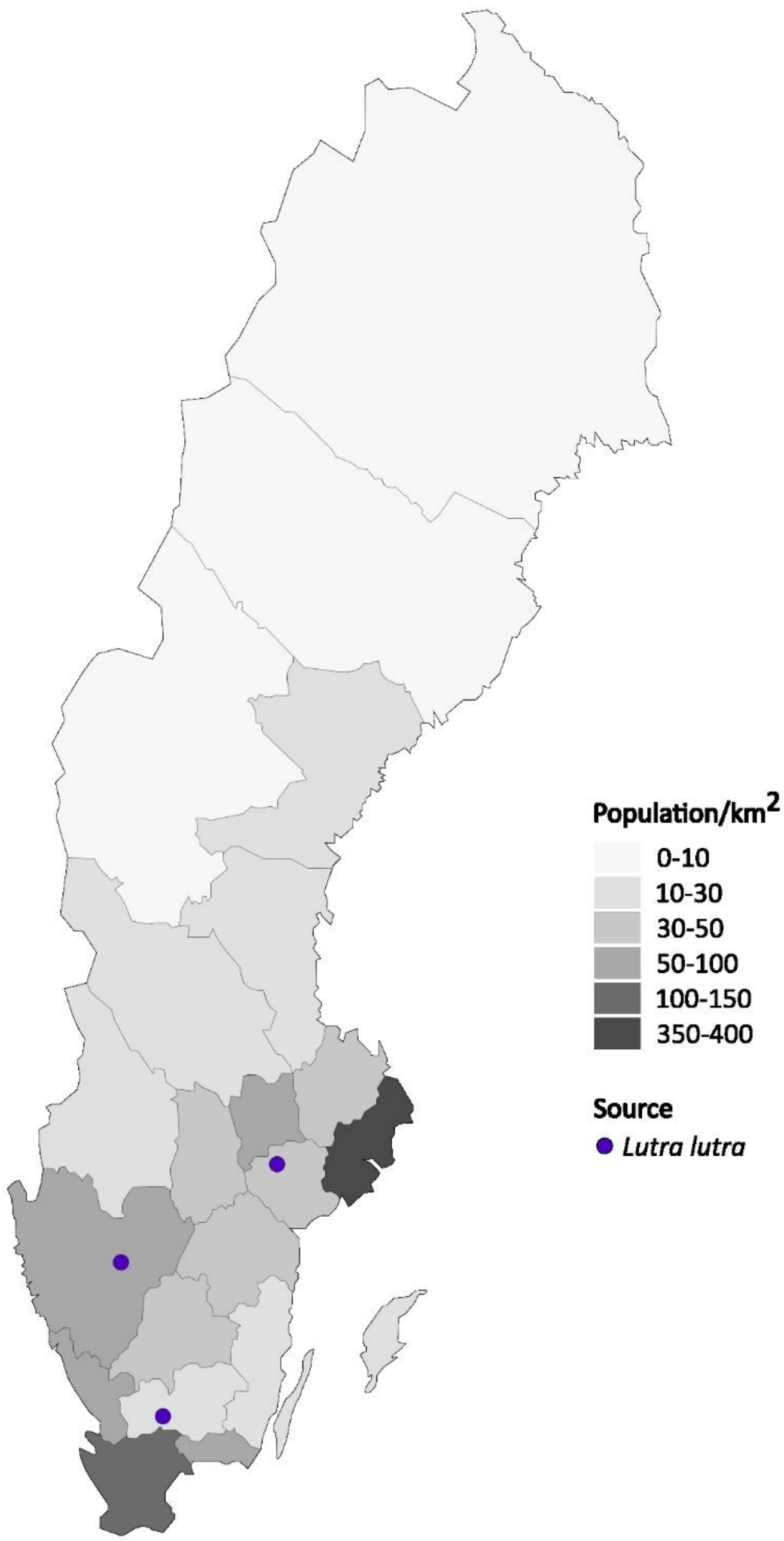

Supplementary Figure S9. Geographical origin of otter samples and human population density per county. Information on geographical origin was not available for five of the samples (not represented in the figure).
